# Meta-analyses of randomized controlled trials assessing the effect of digital tools on step count and moderate-to-vigorous physical activity in healthy children and adolescents

**DOI:** 10.3389/fdgth.2026.1701301

**Published:** 2026-06-04

**Authors:** Garden Tabacchi, Roberta Cottone, Antonino Scardina, Marta Giardina, Antonella Amato, Sonya Vasto, Giulia Accardi, Valentina Di Liberto, Monica Frinchi, Paolo Boffetta, Walter Mazzucco, Marianna Bellafiore

**Affiliations:** 1 Department of Psychology, Educational Science and Human Movement, University of Palermo, Palermo, Italy; 2 Department of Neuroscience, Biomedicine and Movement, University of Verona, Verona, Italy; 3 Department of Biological, Chemical and Pharmaceutical Sciences and Technologies, University of Palermo, Palermo, Italy; 4 Department of Biomedicine, Neuroscience and Advanced Diagnostics (BIND), University of Palermo, Palermo, Italy; 5 Department of Medicine and Surgery, University ofi Enna Kore, Enna, Sicily, Italy; 6 Department of Medical and Surgical Sciences, University of Bologna, Bologna, Italy; 7 Department of Health Promotion, Maternal and Infant Care, Internal Medicine and Medical Specialties (PROMISE) University of Palermo, Palermo, Italy

**Keywords:** digital tools, effectiveness, healthy, moderate-to-vigorous physical activity, physical activity, schoolchildren, step count

## Abstract

**Background:**

Digital tools can influence young people's physical activity both positively and negatively. This meta-analysis (MA) aims to determine whether global interventions based on the use of digital tools are effective in increasing step count and moderate-to-vigorous physical activity (MVPA) among healthy school-aged children.

**Methods:**

This MA builds upon a previous umbrella review that identified 43 randomized controlled trials evaluating digital tools aimed at increasing step counts and daily MVPA in healthy children and adolescents aged 6–17 years. Risk of bias was assessed using the revised Cochrane RoB 2 tool. Effect estimates were expressed as weighted mean differences (WMDs) with 95% confidence intervals (CIs). Heterogeneity was assessed using the I² statistic, and *τ*² (tau-squared) was used to calculate prediction intervals. Sensitivity and subgroup analyses were performed, along with an assessment of small-study effects to detect potential publication bias. This study was registered in PROSPERO (CRD42024510602).

**Results:**

Data were extracted from 18 step count and 32 MVPA observations. Although 27.8% of the studies were judged to have a high risk of bias, this did not significantly affect the overall effectiveness of the interventions. The meta-analysis found no significant overall effect of digital interventions on daily step count (WMD: 267.81; 95% CI: −198.58–734.20), but a significant increase in MVPA minutes per day was observed (WMD: 2.72; 95% CI: 0.83–4.61). Subgroup analyses indicated greater effectiveness when the digital component included a wearable device or a combination of tools, a non-digital component was integrated into the intervention design, and the intervention was delivered via a mix of devices.

**Discussion:**

Globally, digital interventions appear to be effective in increasing MVPA among school-aged children but not in significantly increasing step counts. The results of the subgroup analyses indicate that the generalizability of digital interventions remains limited. To enhance effectiveness, future interventions should be carefully tailored, taking into consideration specific factors such as the type of digital tool, the delivery device, and the integration of supportive non-digital elements.

**Systematic Review Registration:**

PROSPERO CRD42024510602.

## Introduction

Regular physical activity (PA) is essential for preventing non-communicable diseases from early life (1). However, over 80% of children and adolescents are physically inactive worldwide (2) and fail to meet the current recommendations for youth aged 5–17 years of at least 60 min of moderate-to-vigorous physical activity (MVPA), defined as any activity with a metabolic equivalent of task (MET) value comprised between 3 and 5.9, and vigorous-intensity physical activity, which corresponds to PA ≥6 METs (3–5). Moreover, recent studies show a decline in MVPA among youth over time (3).

Another common indicator for quantifying PA is represented by daily number of steps (further indicated as “step count”), which incorporates both light and moderate-to-vigorous activities (6). It is commonly accepted that number of steps is associated with various health outcomes in children and adolescents, such as risk of mortality and cardiovascular disease (7), BMI, waist circumference, body fat percentage, and cardiorespiratory fitness (i.e., VO_2_max) (8); thus, this behavior could help maintain an optimal level of health. Many interventions aiming at increasing PA in youth have focused on the number of steps per day as an indicator of a correct amount of physical activity (9–11), although there is no official agreement on the ideal number of steps for different age groups. According to the study by Tudor-Locke et al. (12), 60 min of MVPA could be achieved through 13,000–15,000 steps per day in male children, and 11,000–12,000 steps per day in female children, while 10,000–11,700 steps are needed for both adolescent boys and girls. A systematic review stated that young people between the ages of 5 and 19 years old should get 12,000 steps per day (13), and the same evidence was provided by another study by Colley et al. (14). In everyday practice, the majority of pedometers provide guidelines as per the 10,000-step protocol or sometimes reduce the goal to 7,500 steps daily, indicating as “sedentary” an amount less than 5,000 steps per day.

The main determinants of these low physical activity levels in children and adolescents are a mix of personal, social, and environmental factors, with the strongest and most consistent evidence pointing to intention, self-efficacy, planning, support from others, age, and some sociodemographic factors (15). For children, the clearest prospective determinant identified in the evidence was intention to be physically active (16). For adolescents, evidence pointed more often to age, ethnicity, and planning, while other studies found that higher perceived behavioral control, support for physical activity, and self-efficacy were linked to smaller declines in activity (17). At a broader level, inactivity is also associated with social and environmental influences, including parents who are inactive, and a range of social, environmental, economic, and sociocultural factors that affect sports participation (18, 19).

Different interventions have been implemented in the last few decades to address these determinants, and many of them were based on digital tools. The most common digital tools used in this age range to deliver the intervention included gamification components, apps, text messages, and websites. However, discrepancies in the effectiveness of such digital tools have emerged from trials on PA available from the international literature. The use of digital technologies targeted to youth has been suggested worldwide as a tool for disease prevention (20), and, in particular, to increase PA in children and adolescents and improve unhealthy lifestyles that can lead to obesity and chronic diseases (21, 22).

Digital interventions are theoretically expected to work by changing these determinants through behavior change techniques. Common theoretical pathways include increasing intention and motivation through tailored messages and reminders; improving self-efficacy and perceived control through feedback, coaching, and achievable goals; adding social connection through online interaction, peer support, or coach contact; and reducing access barriers by delivering activity support at home or in flexible formats that fit adolescents’ schedules and contexts (15, 23, 24). For example, self-monitoring through pedometers or accelerometers allows users to track steps or activity minutes, increasing awareness of sedentary behavior. Feedback loops and real-time progress notifications reinforce activity, while goal setting provides clear benchmarks that enhance motivation and self-efficacy. Gamification and social features, such as points, badges, and challenges, make activity engaging and foster intrinsic and extrinsic motivation. Finally, prompts and reminders serve as behavioral cues to interrupt inactivity. By linking these digital components to mechanisms of awareness, motivation, reinforcement, and habit formation, such interventions are theoretically positioned to promote sustained increases in steps and MVPA, though their effectiveness depends on which pathways are successfully engaged.

Recent evidence suggests that digital interventions (e.g., mobile apps, wearables, and web-based programs) lead to small-to-moderate improvements in physical activity, particularly when they include behavior change techniques such as goal setting and feedback. However, their effectiveness is highly variable across populations and intervention types, and overall effect sizes remain modest. This could be ascribed to the different settings considered, follow-up, population targets, or tools, methods, and procedures used (25). Some studies used self-reported measures, such as questionnaires, diaries, or activity logs, that are easy to collect but provide more biased data; other studies used objective approaches, like device-based measures collected through tools, such as wearables, that can be cheap and provide accurate and reliable data on PA, assisting with behavior change or self-monitoring (26, 27). The different tools used to measure PA outcomes objectively were mainly activity trackers and wearables (28). The literature is also limited by methodological weaknesses, including short follow-up periods, risk of bias, and declining user engagement over time; in addition, inequalities in access and use may influence outcomes (29).

Despite a growing body of research on digital interventions, important gaps remain. In particular, there is limited quantitative synthesis of studies evaluating the global effect of wearable-based interventions on objective physical activity outcomes, such as step count and MVPA, especially among youth. Moreover, existing studies often focus on effectiveness without adequately examining the underlying behavioral mechanisms through which these interventions may counteract physical inactivity, such as motivation, self-regulation, social influence, and environmental constraints.

From a theoretical perspective, digital components, including real-time feedback, goal setting, and activity tracking, are expected to influence physical activity by enhancing self-monitoring, reinforcement, and behavioral awareness, which in turn may lead to increases in daily steps and MVPA. However, the extent to which these mechanisms translate into measurable and sustained behavioral changes remains unclear.

Taken together, although digital interventions show promise for promoting physical activity, the evidence base is fragmented, variable in quality, and difficult to synthesize across heterogeneous designs and populations. This underscores the need for a rigorous and up-to-date meta-analysis to quantify overall effects, explore sources of heterogeneity, and provide clearer guidance for research and practice. Addressing these gaps through a rigorous meta-analysis (MA) will provide a more precise estimate of intervention effects and help clarify both their effectiveness and the pathways through which they operate.

To provide a clearer research landscape and fill this knowledge gap, we conducted a meta-analysis to assess whether worldwide digital-based interventions are effective in increasing step count and MVPA among healthy children and adolescents.

## Methods

### Study design

This MA was conducted on randomized controlled studies (RCTs) that were selected in a previous systematic review (SR) of reviews and meta-analyses (30). The review is registered in PROSPERO (CRD42024510602), where full methodological details are available. The identification of RCTs followed a two-phase process: first, systematic reviews and meta-analyses were selected through a systematic review; second, these reviews were screened to identify RCTs that met the predefined eligibility criteria.

A manual search was conducted alongside the main search by screening the reference lists of included reviews and consulting relevant websites and grey literature sources. For articles not accessible online, the authors were contacted to request full-text copies.

The work was conducted according to rigorous, peer-reviewed protocols designed to ensure an unbiased assessment of the evidence. This process involved comprehensive literature searches, critical evaluations of study quality, and a quantitative synthesis of findings to generate clear, evidence-based conclusions.

### Eligibility

Study eligibility was determined based on the Population, Intervention, Comparison, Outcome, and Study type (PICOS) criteria.

Population: Healthy individuals aged 6–17 years, including overweight/obese participants without diagnosed diseases.

Intervention: Digital approaches to increase physical activity, including devices, wearables, apps, social media, messaging tools, web-based platforms, and gamified or coached programs.

Comparison: Any intervention or no intervention.

Outcome: Physical activity as a primary or secondary outcome.

Study type: Systematic reviews and meta-analyses of RCTs. Reviews without PA outcomes, non-digital interventions, measurement-only tools, or targeting specific or clinical populations were excluded.

### Search strategy

The search was performed by following the PRISMA-S (Preferred Reporting Items for Systematic review and Meta-Analysis literature search extension) checklist (31). Five databases were explored: Scopus (Elsevier), PubMed/MEDLINE (NCBI), Web of Science (Clarivate), the Cochrane Database of Systematic Reviews via the Cochrane Library (Wiley), and SPORTDiscus via EBSCOhost. Publications were limited to the years after 2017. This timeframe was selected based on evidence that studies on second-generation technologies (e.g., smartphones and wearable devices) have increased markedly since 2013 (32); consequently, a review published from 2018 onward would likely capture a broader range of these tools.

Search keywords combined terms related to digital tools and physical activity outcomes, including apps, wearables, social media, messaging, exergames, digital assistants, and devices such as pedometers or accelerometers. No language restrictions were applied.

Two independent reviewers screened titles and abstracts first, followed by full texts. Disagreements were resolved through discussion or, if needed, consultation with a third reviewer.

A total of 62 RCTs assessing the effect of digital interventions on PA among schoolchildren were extracted from the selected reviews. Studies presenting measures of step counts and of MVPA as outcomes were further selected, resulting in a total of 43 trials that were included in the MA (33–77).

### Data extraction

Step count was defined as the daily average number of steps, and MVPA was defined as the daily average of minutes of moderate-to-vigorous activity. Data on step count and MVPA, with their standard deviations (SDs), were extracted by two independent authors, and discrepancies were resolved through the intervention of a third.

The following information was first registered on an Excel database and then exported to the statistical software for the analyses: authors; publication year, classified into two categories (<2018 and ≥2018); country (Europe, USA, Australia/New Zealand, Asia); type of intervention; focus task of the intervention (PA only, PA and other outcomes, weight, other tasks such as health, diet, etc.); theoretical foundation; setting of implementation (home, school, and community other than school: hospital, clinics, military service); school age of the target population (elementary, middle, high); special populations considering different weight status (underweight, overweight, obese), physically inactive, or samples with only males or females; sample dimension; follow-up duration (<9 weeks, 9–20 weeks, >20 weeks); number of intervention arms; intervention group (IG) and control group (CG) treatments; type of digital component for intervention delivery (gamification, app, text messages, web, social media, wearable, mix of the previous); digital device type with the model for intervention delivery (pedometer, accelerometer, console, computer, smartphone, etc.); use of non-digital components for intervention delivery (e.g., incentives, goals, etc.); outcome measure (number of steps or MVPA); and means and SDs of the step count and of MVPA both for the IGs and CGs.

### Data analysis

Meta-analyses were performed separately for the data on steps and MVPA.

Since all selected data were derived from objective measurement tools, we used the mean differences weighted by the inverse of variance (WMD) as the outcome measure to estimate the overall effect.

Initially, a fixed effects model using the method of Mantel and Haenszel was run; when the assumption of study homogeneity was not reasonable for our data (due to high heterogeneity of the studies), a random effects model using the method of DerSimonian and Laird was performed (78), with weights inversely related to the total variance.

Heterogeneity was estimated using the I-square statistic (I^2^), which is the percentage of the total variability in a set of effect sizes due to true heterogeneity. Different metrics have been used in the literature to define the level of heterogeneity (low, moderate, and high) (79, 80); in the present study, that of Deeks et al. (80) was considered, proposing 0%–40% as non-important heterogeneity, 30%–60% moderate heterogeneity, 50%–90% substantial heterogeneity, and 75%–100% considerable heterogeneity. Heterogeneity was explained through subgroup and sensitivity analyses. However, only using the I^2^ was not considered sufficient, since it is a proportion rather than an absolute value; this means that it could give information on what proportion of the observed variance is likely to remain if we could somehow remove the sampling error (81), but does not inform on how much the effect varies between studies. Thus, the tau-squared (*τ*^2^) statistic, which is the SD of the between-study variation on the scale of the original outcome, was further calculated (80) to estimate the prediction interval (PI). A PI is the “interval within which the effect size of a new study would fall if this study was selected at random from the same population of the studies already included in the meta-analysis” (82). Therefore, in order to better predict the impact of between-study heterogeneity, alongside the summary effect size and the 95% confidence interval (CI), PIs of the overall estimate were obtained; they were undefined if fewer than three studies were included in the subgroup analysis.

Possible outliers were identified through the leave-one-out method, which performs multiple meta-analyses by excluding one study at each analysis, thus detecting the infl\uence of each study on the overall effect-size estimate.

As suggested by Sterne et al. (83), a small study effect was estimated to identify the potential tendency of the intervention effect to be more beneficial in smaller studies. This was performed using funnel plots, displaying the standardized mean differences (SMDs) on the *x*-axis and the standard error of the SMDs on the *y*-axis. When funnel plot asymmetry was detected, this possibly explained publication or other reporting biases using Egger's linear regression test (84). The Duval and Tweedie non-parametric “trim-and-fill” method was also used to account for publication bias and estimate the number of unpublished studies (85); this is a simple way to handle missing studies in a meta-analysis, as it identifies missing values and recalculates the effect size, thus helping correct for publication bias.

STATA/MP 12.1 (Stata-CorpLP, College Station, TX, USA) was used for the statistical analysis, with the specific commands “metan” for the meta-analysis, “metabias” for small study effect, and “metatrim” for the trim-and-fill method.

### Quality and risk of bias

The risk of bias of each study was assessed using the five dimensions of the revised Cochrane Rob 2 tool for randomized trials (86). The classification of individual studies was based on an algorithm that calculated the risk of bias in the various domains, with studies classified at “low risk,” “some concerns,” or “high risk.” This assessment was performed by two authors independently, with a third author resolving possible disagreements.

## Results

### Study characteristics

Out of the retrieved 43 studies, a total of 19 RCTs reported step counts and/or MVPA as mean number/day (and SD) and/or mean minutes/day (and SD), respectively (Supplementary Material 1) (33–37, 41, 44, 49, 51, 53–55, 58, 61, 68, 69, 73–75). A total of 10 studies relied on foundation theories, such as the Theory of Planned Behavior, the Self-Determination Theory, the Social Cognitive Theory, and the Transtheoretical Model of Behavior Change. (87–89).

**Figure 1:**
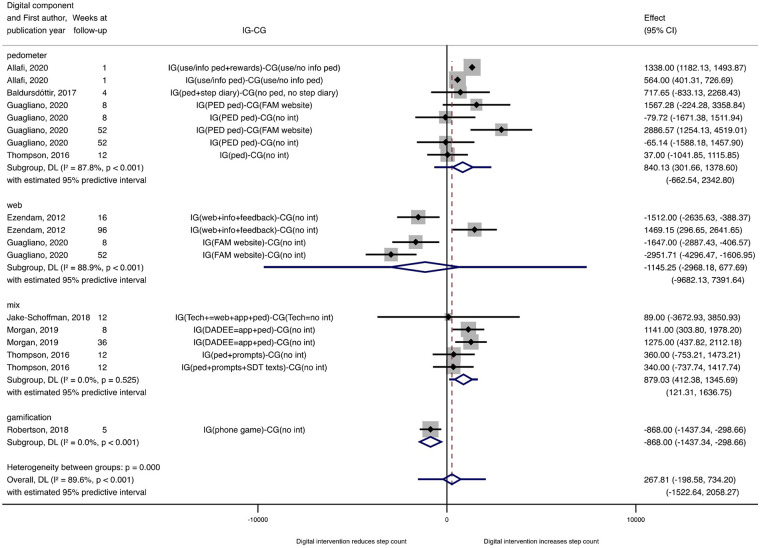
Frequencies of the RCT characteristics of studies measuring step counts and studies measuring MVPA. *For steps: diet; for MVPA: diet, weight, cardiorespiratory fitness, and psychological aspects. ^§^For steps: energy balance and sleep; for MVPA: health, obesity, and body composition. ^For steps: website + app + text messaging, wearable + website, and app or text messaging; for MVPA: wearable + website, text messaging, or social media and gamification + website + wearable. ^#^For steps: computer + wearable and smartphone + wearable; for MVPA: smartphone + iPod touch, computer + wearable, and smartphone/mobile phone + wearable. ^†^For steps: goals + incentives, PA advice + step diary, program for activities, goals + rewards; group education + practical sessions, daughters resources (tasks, folders), sports skills program, and sport equipment pack; for MVPA: incentives + education + social networking/forums/messaging + program for activities; goals + rewards, school program, information about increasing PA, healthy eating, or weight loss, encourage to meet current PA recommendations, booklet with instructions, calculate compliance and adherence, and pediatric weight management program.

Figure 1 shows the frequency distribution of the characteristics of the RCTs that assessed step counts (SC studies) and/or MVPA (MVPA studies).

The majority of the SC studies were recently published (62.5%), while the MVPA studies were published before 2018 (66.7%); both types of studies were conducted mainly in Europe (37.5% and 26.7%, respectively). The SC studies had PA as the primary and unique focus task (62.5%), while the MVPA studies focused mainly on PA and other variables, such as diet, weight, cardiorespiratory fitness, and psychological aspects. Half of the SC studies were conducted at school (50%), while a majority of the MVPA studies were conducted at home (53.3%). The main target population was children from elementary and/or middle school (75% and 66.7%). Their follow-up was mostly brief, i.e., 8 weeks or less for 62.5% of the SC studies and 53.3% of the MVPA studies. The main digital component used to deliver the SC interventions was a mix of tools (50%), such as website + app + text messaging, wearable + website, app or text messaging, followed by the use of only a wearable (37.5%), which was a pedometer in general (25%); as a consequence, the main digital devices used for delivering the interventions were composed of a mix of tools, such as computer + wearable or smartphone + wearable. For the MVPA interventions, the preferred digital component used was gamification (60%), which included exergames, role-playing videogames, and games included in immersive apps; this was followed by the use of a mix of tools (40%), i.e., wearable + website, text messaging, or social media, gamification + website + wearable. The most used device models were Yamax pedometers (50%) for steps and Actigraph accelerometers (80%) for MVPA. A non-digital component was added in 50% of the SC studies and consisted of goals, incentives, PA advices, step diaries, program for activities, rewards, group education, practical sessions, resources as tasks or folders, sports skills programs, and sport equipment packs; a similar proportion was found for the MVPA studies (46.7%) for the addition of incentives; education sessions; social networking/forums/messaging; program for activities; goals; rewards; school programs (sport, interactive seminars, nutrition workshops, lunch-time PA sessions, PA and nutrition handbooks, and parent newsletters); information about increasing PA, healthy eating, or weight loss; encouragement to meet current PA recommendations; booklet with instructions; calculating compliance and adherence; and pediatric weight management programs (sessions on foods and drinks, reduction of screen time, goal setting, and increasing PA). The trials were mainly organized with a two-arm design (62.5% for the SC studies and 73.3% for the MVPA studies), with CG treatment represented by no intervention in the majority of cases (75% and 60%, respectively).

### Risk of bias assessment results

**Figure 2:**
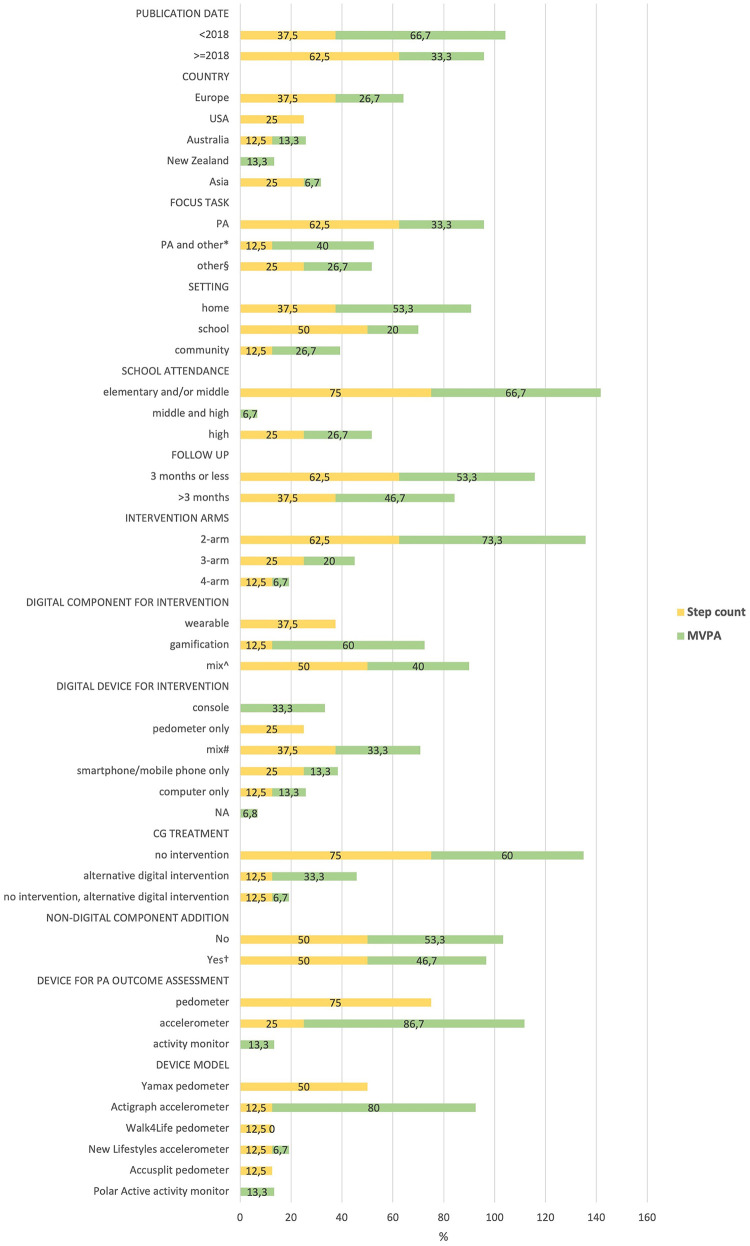
Risk of Bias 2 (RoB-2) results in percentages.

Figure 2 shows the risk of bias assessments of the included 43 studies. A total of 31.9% of the studies showed a low overall risk of bias (41, 46, 47, 49, 64, 71, 74), while some concerns were found for 40.3% of the included studies (33–37, 43, 45, 48, 50, 51, 54, 60, 61, 65, 67, 69, 73, 76, 77) and 27.8% had a high risk of bias rating (38–40, 42, 44, 52, 53, 55, 56, 58, 63, 66, 68, 70, 72, 75). The most critical items were possible deviations in the intended interventions and the measurement of the outcome, since participants or assessors were often not blinded. As stated by other authors (90), the limitation of not being able to blind staff and participants derives from the nature of the intervention, since a device to measure PA needs to be worn by the participants.

### Step count meta-analysis

Eight RCTs focusing on step count measures were selected for the MA (33, 34, 44, 49, 51, 61, 68, 74). Considering that different follow-up periods were indicated and/or different intervention groups were tested against a control group in each study, a total of 18 observations was obtained from these eight RCTs and included in the MA.

Average daily step count across studies ranged between 2,655 and 11,892. The combination of the retrieved studies allowed for a population sample of 1,723 to be obtained (879 for the IG, 844 for the CG).

After applying a fixed effects model in the MA, a positive overall effect was found, with step number significantly increasing in the IG compared to the CG (WMD 833.06, 95% CI: 728.88–937.25), even though high heterogeneity was present (I^2^ 89.6%, *p*-value = 0.000). Considering the presence of heterogeneity between the studies, a random effects model was used, but it did not show overall effectiveness in the IG over the CG (WMD 267.81, 95% CI: −198.58–734.20).

After running subgroup analyses, the publication date, follow-up period, school age, setting, device for measurement, and the overall risk of bias did not explain the heterogeneity, and differences were not observed between the subclasses in the increase in number of daily steps.

**Figure 3:**
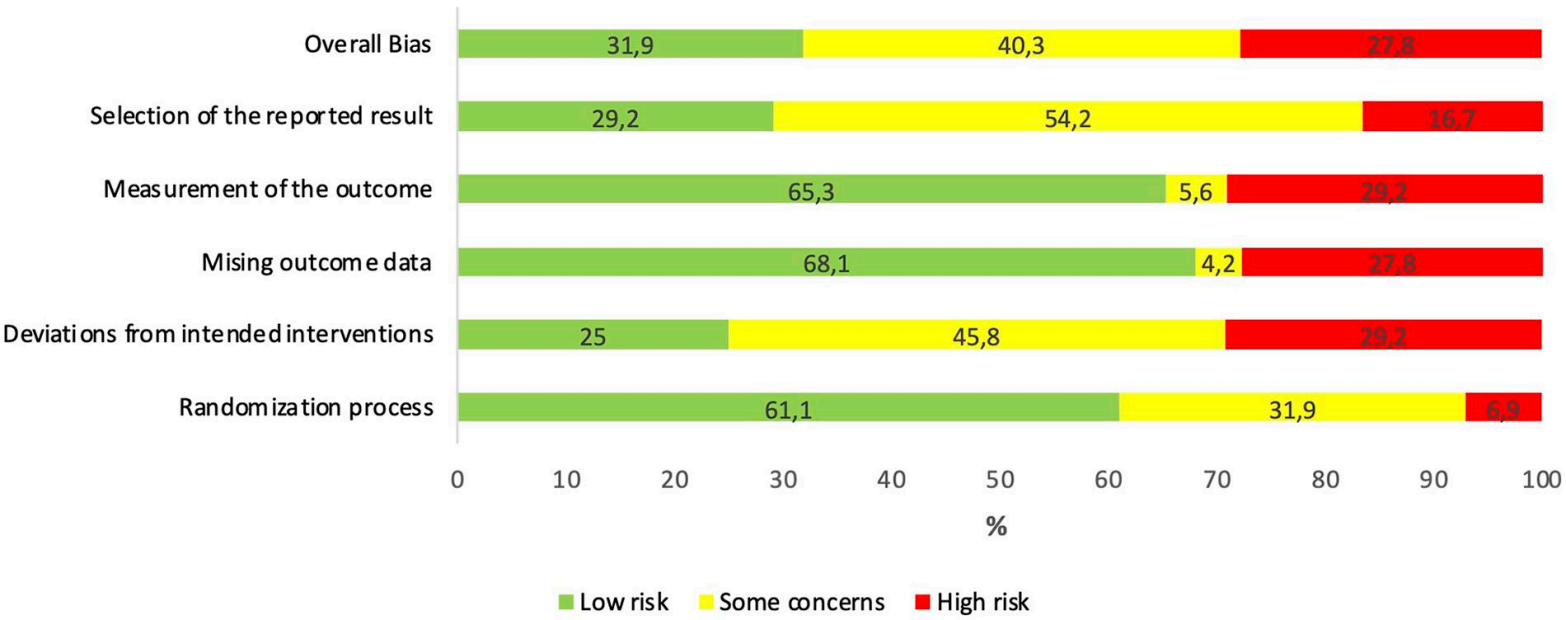
Forest plot of the subgroup analysis of the 18 retrieved RCTs by the digital component used to deliver the intervention to increase step count. PIs are shown together with 95% CIs. CG, control group; DL, Der Simonian–Laird estimate; IG, intervention group; int, intervention; ped, pedometer; SDT, Self-Determination Theory.

A positive pooled effect, instead, was found when a pedometer (WMD 840.13, 95% CI: 301.66–1,378.60; PI: −662.54–2,342.80) or a mix of components (including pedometers) were used as an intervention delivery method, such as pedometer together with the Internet, an app, or text (WMD 879.03, 95% CI: 412.38–1,345.69; PI: 121.31–1,636.75); in this last case, the heterogeneity was annulled (I^2^ 0.0%, *p*-value = 0.525) and the PI did not cross the null line (Figure 3). Interventions were also found to be effective when non-digital components were added to digital ones (WMD 754.22, 95% CI 184.38–1,324.05; PI: −1,136.75–2,645.18), in regions such as Asia and Oceania (WMD 951.37, 95% CI: 192.86–1,709.87, and WMD 1,208.00, 95% CI: 616.02–1,799.98, respectively), and when the Yamax pedometer model was used for step outcome measurement (WMD 789.91, 95% CI: 261.28–1,318.55; PI: −849.14–2,428.96) (Supplementary Material 2).

Since the use of gamification was reported in just one study and was not effective, and also the use of the Internet to deliver the intervention was not effective, we decided to deeply investigate the 13 observations that resulted in effectiveness, in which a pedometer or a mix of tools that included a pedometer was used (33, 34, 49, 51, 61, 74), thus excluding the studies of Ezendam et al. (44) and Robertson et al. (68) and two observations from Guagliano et al. (49).

The random effects model found a significant increase of 831.62 steps (95% CI 425.11–1,238.16; 95% PI: −366.49–2,029.73), even though heterogeneity was high (I^2^ 80.2%).

**Figure 4:**
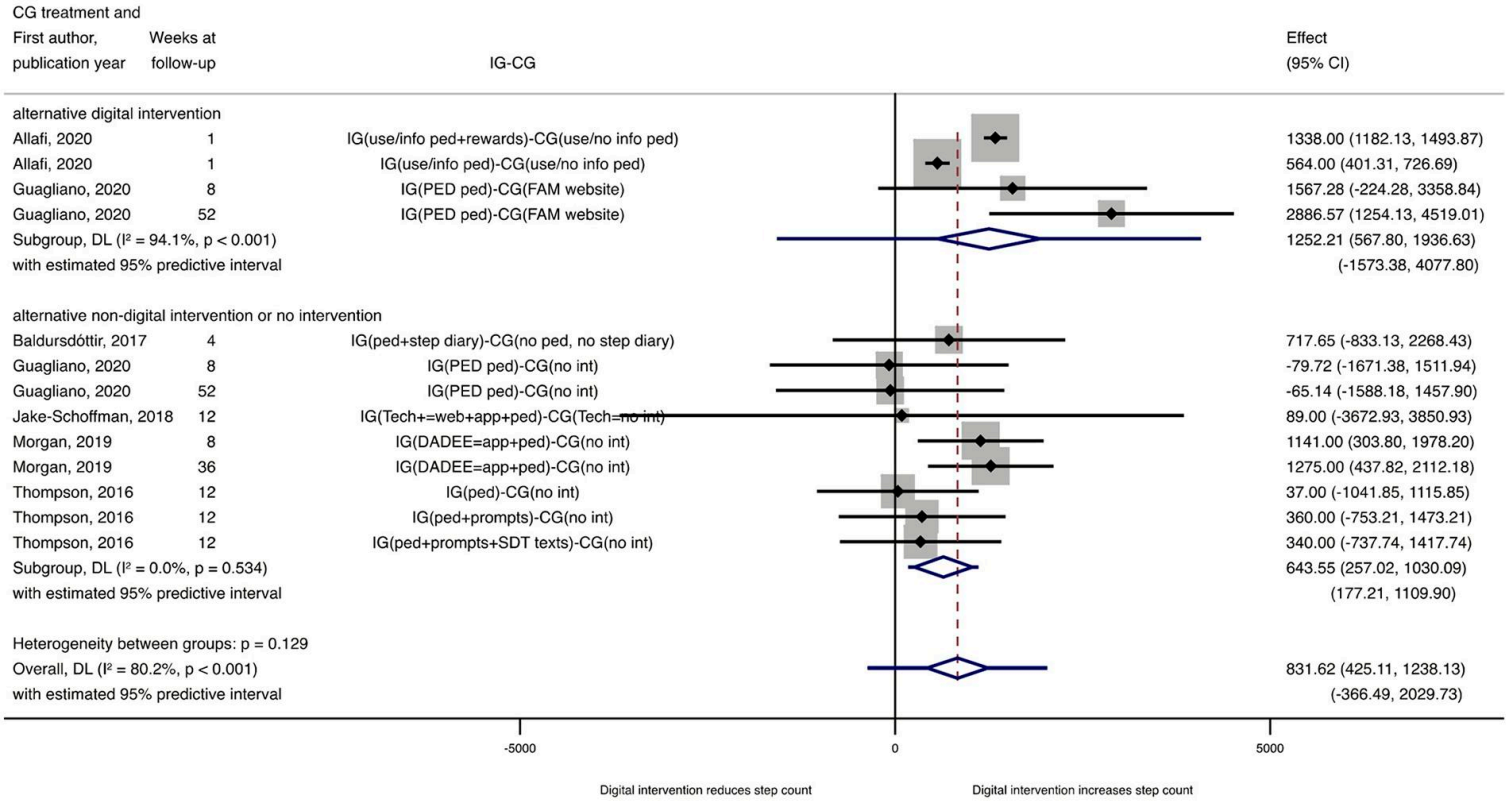
Forest plot of the subgroup analysis of the 13 RCTs using wearables and a mix of tools for intervention delivery to increase step count, by control group treatment. PIs are shown together with 95% CIs. CG, control group; DL, Der Simonian–Laird estimate; IG, intervention group; int, intervention; ped, pedometer; SDT, Self-Determination Theory.

When subgroup analyses were performed, the effect was larger when an alternative digital intervention was used in the CG (WMD 1,252.21, 95% CI: 567.80–1,936.63; 95% PI: 1,573.38–4,077.80). In RCTs that used an alternative non-digital intervention or no intervention in the CG, an increase of 643.55 steps per day was observed (WMD 643.55, 95% CI: 257.02–1,030.09; 95% PI: −177.21–1,109.90) (Figure 4), and, notably, the heterogeneity was annulled (I^2^ 0.0%, *p*-value = 0.534) and the PI was very short and did not cross the null line.

**Figure 5:**
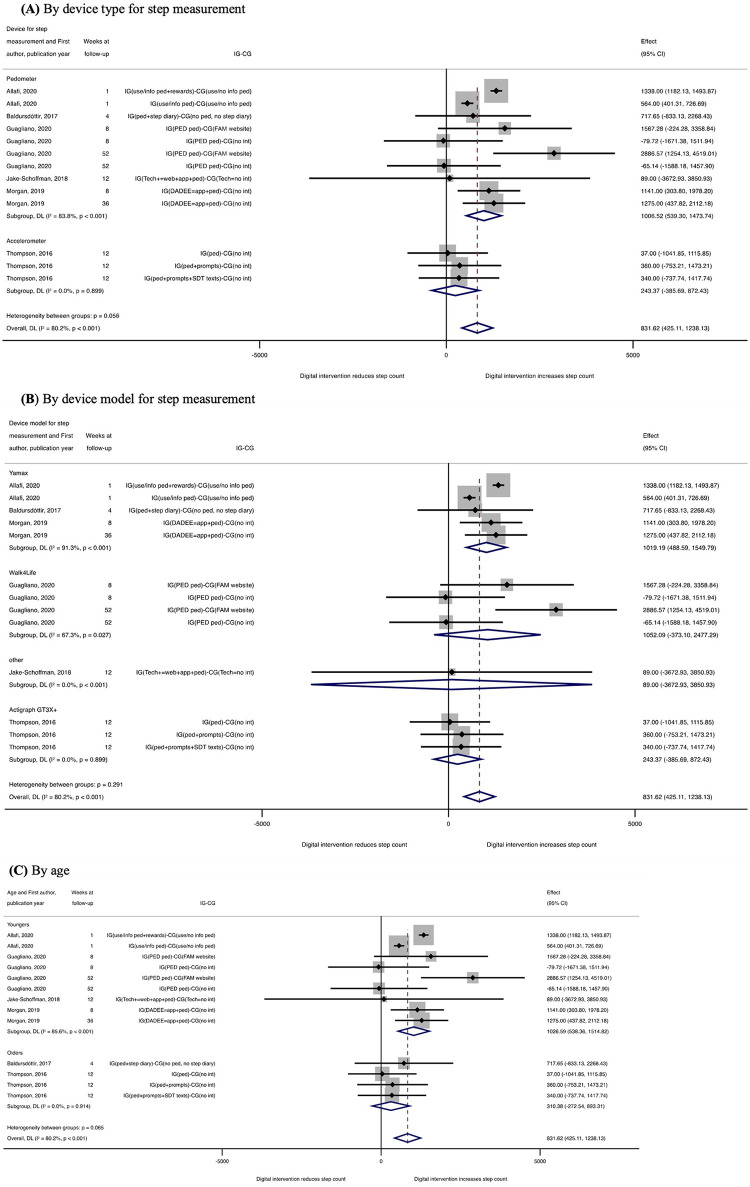
Forest plot of the subgroup analysis of the 13 RCTs using wearables and a mix of tools for intervention delivery to increase step count, by device type **(a)** and model **(b)** for step measurement, age **(c)**, and follow-up CG, control group; DL, Der Simonian–Laird estimate; IG, intervention group; int, intervention; ped, pedometer; SDT, Self-Determination Theory.

In interventions in which pedometers were also used as step-measuring devices (beyond being used to deliver the intervention), the children in the IG had a significant increase of 1,006.25 steps (95% CI: 539.30–1,437.74; PI: −292.08–2,305.11) compared to the CG, while the studies that used an accelerometer were ineffective (Figure 5). A detailed investigation on the model of the devices evidenced how the Yamax model was the only effective model (WMD 1,019.19, 95% CI: 488.59–1,549.79; PI: −784.75–2,823.13) compared to the Walk4life and ACCUSPLIT pedometers or the Actigraph accelerometer (Figure 5). Interventions that were targeted to younger pupils were effective in increasing steps by 1,026.59 (95% CI: 538.36–1,514.82; PI: −333.48–2,386.66), even though these studies were quite heterogeneous. Studies conducted on older students were found to be ineffective but homogeneous (I^2^ 0.0%, *p*-value = 0.000, PI: −969.30–1,590.07) (Figure 5). The same results were obtained in the subgroup analysis of the publication year, since the subgroups were composed of the same RTCs as the age group subgroup analysis. The studies that were published more recently were more effective compared to those published before 2019. Interventions were more effective in the short term (12 weeks or less) (WMD 719.45, 95% CI: 271.80–1,167.11; PI: −536–1,975.09) compared to the long-term measurements, which did not show any step increase (Figure 5).

A table summarizing the PIs for the RCTs that showed effects on step count and MVPA is presented in Supplementary Material 3.

### Small study effect in studies assessing step counts

**Figure 6:**
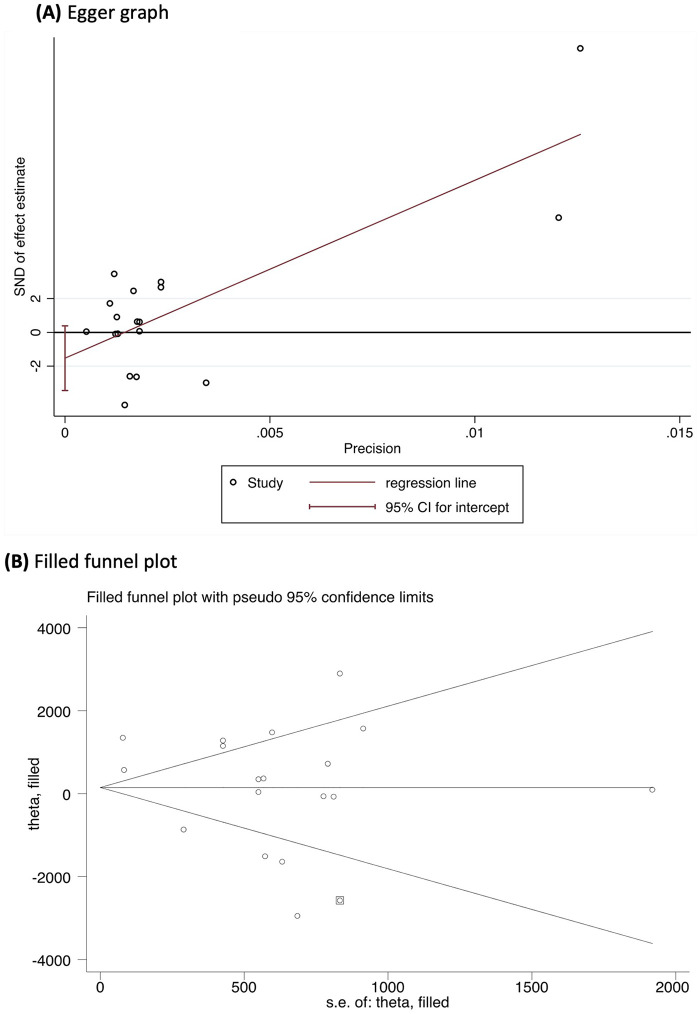
Egger graph **(A)** and filled funnel plot **(B)** of the 18 observations of the step-count studies included in the MA. The filled funnel plot was obtained using the trim-and-fill method; the empty circles represent the included observations, while an empty circle surrounded by a square represents a missing observation.

A small study effect was not found (Egger's test coefficient: −1.527, SE 0.905, *p*-value: 0.111) and no asymmetry was present in the funnel plot (Figure 6). The trim-and-fill method showed that one study was missing at the bottom left of the funnel plot to obtain symmetry (Figure 6). The recalculated pooled estimate after including the missed study was not significantly different (819.2, 95% CI: 715.3–923.2). These results suggested that there was no publication bias.

### MVPA meta-analysis

Overall, 32 measures extracted from 15 RCTs were analyzed, for a total of 3,436 people (35–37, 41, 49, 51, 53–55, 58, 68, 69, 73–75).

In the fixed effects model analysis, an overall effectiveness of digital interventions in increasing MVPA was found (WMD 3.40, 95% CI: 2.68–4.13), but high heterogeneity was found (I^2^ 76.6%). The random effects model of MA incorporated heterogeneity, showing a significant overall increase in minutes of MVPA after the digital interventions (WMD 2.72, 95% CI: 0.83–4.61) and a large PI (95% PI: −5.44–10.88).

We first tried to identify outliers by checking that the intervention's confidence interval did not overlap with the confidence interval of the pooled effect, but since seven outliers were identified based on this criterion, we did not consider it correct to exclude all of them. Second, we omitted the two studies from Guagliano et al. (49) that were the only ones that revealed a pooled effect favorable to the control (they used the web-based family tool for the intervention). This omission led to an increase in daily MVPA of 0.62 min (from 2.72 to 3.34 min), an amount that we considered insufficient to justify the possible exclusion of these two observations from the meta-analysis, also considering that the quality of the study was high.

Since the primary objective of this study was to detect whether digital interventions are effective in comparison to non-digital interventions, we tried to identify through sensitivity analyses which kind of digital tool and what CG treatment was considered to better understand the degree of contribution of each study to the effect.

**Figure 7:**
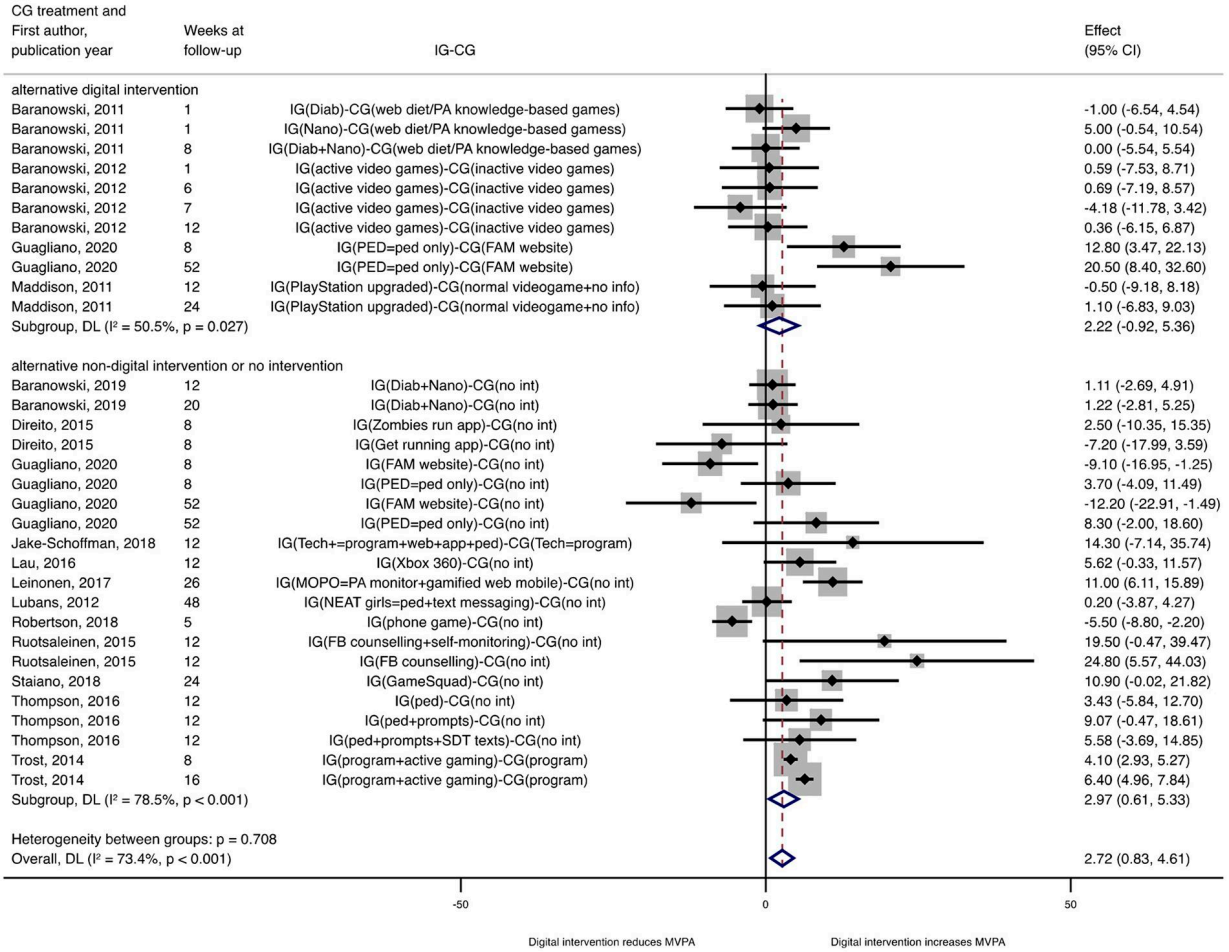
Forest plot of the subgroup analysis of the 32 observations from the RCTs assessing the effectiveness of digital interventions on MVPA increase, by CG treatment. CG, control group; DL, Der Simonian–Laird estimate; IG, intervention group; int, intervention; ped, pedometer; SDT, Self-Determination Theory.

Thus, a first subgroup analysis of CG treatment was conducted, and when an alternative non-digital intervention or no intervention was used in the CG, the overall effect was an increase in MVPA (WMD 2.97, 95% CI: 0.61–5.33, 95% PI: −5.68–11.62), while using a digital tool in the IG was not effective compared to the use of an alternative digital component (WMD 2.22, 95% CI: −0.92–5.36, 95% PI: −6.91–11.35) (Figure 7). For example, using active videogames in the IG compared to non-active videogames or web-based games in the CG (35, 36), or using a PlayStation in the IG compared to regular videogames in the CG (58), did not increase overall effectiveness. In contrast, interventions involving gamification, apps, wearables (e.g., pedometers), or a combination of tools, compared to CGs receiving no intervention, such as in the MOPO, NEAT, and other programs (37, 41, 49, 53–55, 68, 69, 74), or to CGs treated with a non-digital tool (51, 75), were effective in increasing MVPA.

Effective results were also highlighted in the following subgroup analyses (Supplementary Material 4). Studies from Australia and New Zealand were more effective (WMD 3.11, 95% CI: 0.74–5.48; 95% PI: −3.04–9.26) compared to those from European or American countries. Interventions targeting high school students were significantly more effective than those targeting younger children, with an average increase of approximately 4–5 min/day of MVPA (WMD 4.53, 95% CI: 0.35–8.70; 95% PI: −7.93–16.98). The sensitivity analysis showed that in the two studies using web-based interventions (Web FAM) compared to no intervention in the CG, MVPA levels were actually reduced in the intervention group, and these studies involved younger children. When they were excluded, the overall MVPA increase rose by almost 3.5 min.

Digital interventions were efficient when they specifically targeted normal-weight people (WMD 3.44, 95% CI: 0.76–6.11; 95% PI: −6.06–12.94), but high heterogeneity was found (I^2^ 81.3%, *p*-value < 0.001). The RCTs focusing on overweight/obese people only were not effective overall; however, these studies showed low heterogeneity (I^2^ 29.6%, *p*-value = 0.164) and a 95% PI of −4.47–7.68. The effect disappeared for studies conducted in a school environment compared to the home setting (WMD 2.50, 95% CI: 0.12–4.88; 95% PI: −6.96–11.96). Efficacy was found in trials with a medium follow-up between 9 and 20 weeks (WMD 4.27, 95% CI: 1.68–6.87; 95% PI: −2.53–11.07) and not in those with a short (8 weeks or less) or a long duration (20 weeks or more). When the digital device for intervention delivery was a console, an overall effectiveness was evidenced (WMD 3.59, 95% CI: 1.60–5.59, 95% PI: −1.18–8.37). Effectiveness was also found when a mix of devices were used (WMD 6.42, 95% CI: 0.24–12.60, 95% PI: −14.67–27.51). Examples include Tech+, which combines programmatic recommendations, email newsletters, a mobile app, pedometer-based self-monitoring, a website for food and step tracking, and apps (51); MOPO, which uses a wrist-worn PA monitor with feedback and access to a gamified web-based mobile platform (54); NEAT (56), which includes sports sessions, seminars, handbooks, pedometers, newsletters, and text messages; facebook-delivered PA counseling and self-monitoring, as used in Ruotsalainen et al. (69); and the pedometer-based intervention with goal prompts and theory-informed messages in the study by Thompson et al. (74). The use of computers and smartphones did not seem to be successful in increasing MVPA. Among the different models used to measure MVPA, the interventions using Actigraph GT3X were effective overall (WMD 3.20, 95% CI: 1.13–5.27, 95% PI: −3.52–9.92) and the same was found for those using the Polar Active (WMD 13.64, 95% CI: 6.58–20.70, 95% PI: −48.24–75.51).

**Figure 8:**
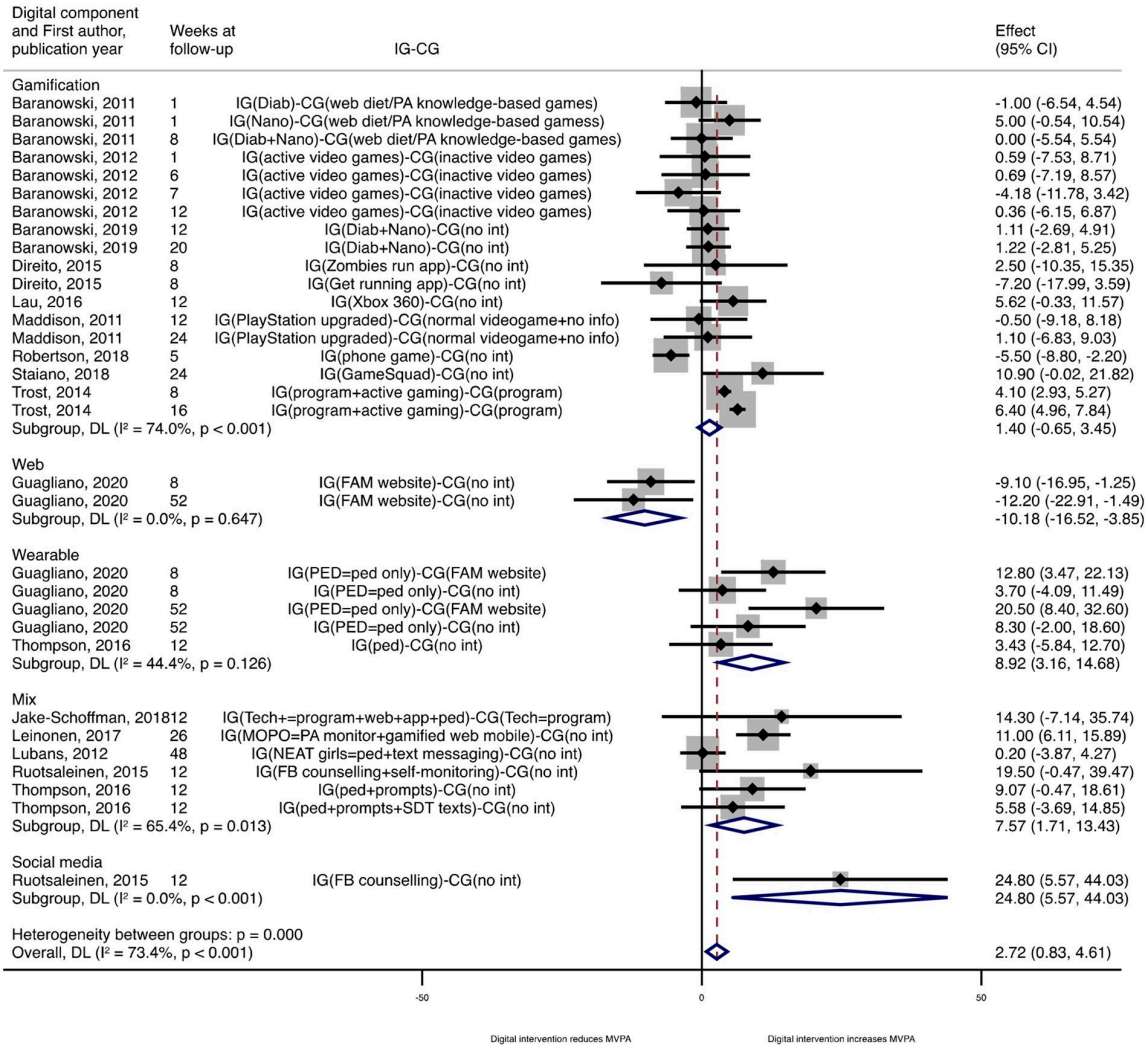
Forest plot of the subgroup analysis of the 32 observations from the RCTs assessing the effectiveness of digital interventions on MVPA increase, by the digital component used to deliver the intervention. CG, control group; DL, Der Simonian–Laird estimate; IG, intervention group; int, intervention; ped, pedometer; SDT, Self-Determination Theory.

Another subgroup analysis was conducted, stratifying the studies by the digital component used to deliver the intervention. This allowed us to highlight how two observations from the study of Guagliano et al. (49), which used a FAM web-based tool, had a contrary effect, i.e., reducing MVPA, in the IG compared to the CG that received no intervention. The use of wearables and of a mix of tools had a large effect in increasing MVPA (WMD 8.92, 95% CI: 3.16–14.68; and WMD 7.57, 95% CI: 1.71–13.43, respectively), with the 95% PIs crossing the null line (−7.81–25.65, and −9.46–24.60, respectively). The use of social media was only investigated in one study (69) and was highly effective (WMD 24.80, 95% CI: 5.57–44.03). In contrast, only gamification was ineffective overall (WMD 1.40, 95% CI: −0.65–3.45) (Figure 8).

This last result induced us to deeply investigate the aspects that could influence the lack of efficacy of this gamification tool in this age range. We further meta-analyzed 18 observations from 9 studies (35–37, 41, 53, 58, 59, 68, 73, 75), adding the study from Leinonen et al. (54) that used a mix of tools, including a gamified portal, for a total of 2,615 subjects. The subgroup analyses of these 19 observations revealed that the lack of efficacy of gamification was not found for a follow-up longer than 20 weeks (WMD 7.97, 95% CI: 1.19–14.40, 95% PI: −62.18–77.77) or in comparison to an alternative non-digital intervention or no intervention in the CG (WMD 3.10, 95% CI 0.29–5.91, 95% PI −5.89–12.09). Moreover, the use of a console as a device to deliver the gamification intervention was efficacious (WMD 3.59, 95% CI: 1.60-5.59, 95% PI: −1.18-8.37) compared with gamified interventions delivered through a computer or smartphone (Supplementary Material 5).

### Small study effect in studies assessing MVPA

The small study effect analysis found funnel plot asymmetry (Egger's test Coeff: 4.86, SE 1,070, *p*-value <0.001), thus indicating a small-study effect (small studies in favor of the control are missing on the left bottom side of the funnel plot) and suggesting publication bias.

**Figure 9:**
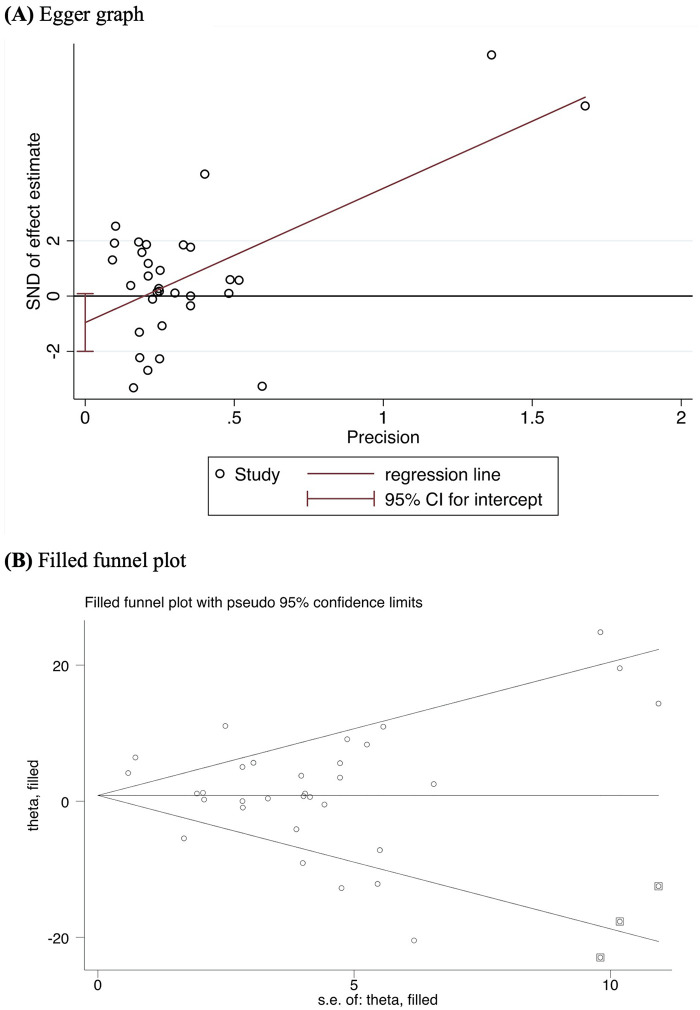
Egger graph **(A)** and filled funnel plot **(B)** of the 32 observations included in the MA of RCTs aimed at increasing MVPA. The filled funnel plot was obtained using the trim-and-fill method; the empty circles represent the included observations, while an empty circle surrounded by a square represents a missing observation.

The trim-and-fill method added three studies that were missing on the bottom left side of the graph, and, after inserting these, the random overall effect was 0.85 (95% CI: −1.176–2.872) (Figure 9).

## Discussion

Our meta-analysis revealed that, on a global scale, digital interventions show a differentiated impact on physical activity outcomes in school-aged populations, appearing to be more effective in increasing MVPA than in promoting overall step count. This discrepancy likely reflects the distinct behavioral mechanisms targeted by such interventions, which often emphasize intensity, structured activity, or short bursts of movement rather than total daily volume. The increase of approximately 3 min per day in MVPA that was found in this study is modest and unlikely to be clinically meaningful at the individual level, particularly for outcomes such as adiposity, cardiorespiratory fitness, or cardiometabolic risk, which typically require larger changes in activity volume or intensity (91, 92). However, two considerations temper this interpretation. First, at the population level, even small shifts in MVPA can translate into a meaningful public health impact when applied across large groups of children. Second, such changes may represent early behavioral movement, especially in predominantly inactive populations, and could be a stepping stone toward larger increases if interventions are sustained or intensified.

A high level of heterogeneity was observed across the studies, highlighting the variability in intervention design, populations, and measurement approaches. While this limits the generalizability of the findings, it also underscores the importance of identifying specific components that may enhance intervention effectiveness.

### Digital tools used for intervention delivery

The subgroup analyses conducted both on the SC and MVPA studies highlighted the crucial role of self-monitoring. Interventions incorporating pedometers and accelerometers appear to facilitate significant daily steps and MVPA increases by reinforcing participants' awareness of their activity levels; this real-time feedback may promote self-regulation and encourage more active choices throughout the day (93, 94). The effectiveness of these tools is further amplified when combined with additional digital components, such as mobile applications, web platforms, or messaging systems. This suggests that multimodal interventions can enhance engagement and reinforce behavioral change through multiple channels, representing a key strategy for achieving health-related goals (95). It is therefore recommended that wearable-based interventions not only be included in future experimental studies but also promoted as a beneficial daily habit for all children.

Contrary to expectations, the gamification component did not consistently lead to greater overall MVPA. However, a closer examination of the studies incorporating gamified elements suggests that these interventions may be more effective in sustaining behavior change over the long term rather than producing immediate increases in MVPA. This pattern appears to differ from the findings reported by Mazeas et al. (96), but is in line with other previous studies (97, 98). A possible explanation is that in the short term, gamified interventions often leverage novelty effects, rewards, and feedback loops to increase motivation and participation, but these mechanisms may not immediately translate into higher MVPA, especially in children and adolescents whose activity levels are also constrained by contextual factors such as school schedules, opportunities for sport, and parental or environmental support. Over time, however, gamification may become more effective by fostering habit formation, intrinsic motivation, and sustained engagement with the intervention platform; as users become familiar with the system, the initial novelty may fade, but persistent behavioral cues (e.g., goal setting, progress tracking, social comparison, or rewards) can support longer-term adherence to PA behaviors (99, 100). This could explain why some studies report more stable or delayed effects rather than immediate increases in MVPA. Additionally, as previously noted, gamified interventions were effective when the CG received no intervention or a non-digital intervention, rather than another digital tool, and when devices other than computers or smartphones were used to deliver the intervention. These aspects should be carefully reconsidered in future interventions involving gamification tools. In particular, the choice of components, such as the CG design, appears to be critical, as interventions using alternative digital tools in the CG seem less effective. Another recommendation would be to increase the number of intervention sessions, allowing the effects to sustain over time.

Currently, the most appealing digital tools for young people include gamification features, wearables, social media, and smartphone apps (98, 101–103). Therefore, when designing interventions targeting this age group, it is essential to align the tools used with their digital preferences.

The observed differences in MVPA outcomes across different device models are likely attributable to methodological and measurement-related factors rather than true differences in intervention effectiveness. The larger effect associated with the Polar Active device (approximately 13.5 min/day) should be interpreted cautiously, as it is based on limited evidence (two trials, three effect estimates) (54, 69), making it particularly sensitive to study-specific characteristics such as intervention design, sample composition, or implementation context. In small datasets, such factors can disproportionately influence pooled estimates and potentially inflate observed effects. In addition, devices differ in how MVPA is operationalized and derived. The Polar Active relies on proprietary, often heart-rate-informed or zone-based algorithms, which may be more responsive to changes in physiological effort and thus yield higher estimates of moderate-to-vigorous activity (104, 105). Conversely, the Actigraph GT3X, which is widely used and extensively validated in pediatric populations, applies standardized accelerometer cut-off points that tend to be more conservative in classifying MVPA (106). Consequently, increases in PA may be partially allocated to lighter intensity categories, resulting in smaller estimated effects (approximately 3 min/day) despite similar underlying behavioral changes. Taken together, these findings suggest that the magnitude of observed effects is influenced not only by intervention efficacy but also by device-specific sensitivity and analytic conventions. Therefore, comparisons across devices should be interpreted with caution, and future studies would benefit from harmonized measurement protocols or calibration studies to improve comparability across accelerometer-based and proprietary monitoring systems.

### Addition of non-digital components

Additionally, studies that incorporated non-digital components, such as goal setting, educational sessions, incentives, or structured activities with peers or guided by teachers, alongside digital tools tended to show greater effectiveness in increasing PA outcomes. Non-digital components likely enhance outcomes by providing accountability, facilitating the understanding and use of digital content, and strengthening motivation and self-efficacy (107). In children and adolescents, social interaction with teachers, parents, and peers plays a central role in shaping adherence, as engagement often depends on external support. These findings suggest that digital tools operate within a broader social context, and their effects may reflect a synergy between technological and human elements rather than purely technological efficacy. Future research should clarify the contribution of human involvement to better inform intervention design.

### Measurement devices (pedometers vs. accelerometers)

In terms of measurement devices, the discrepancy between the higher efficacy in the step increase of interventions using pedometers as a measurement tool compared to accelerometers may be due to differences in how the data are exported and processed, sometimes automatically via the cloud, and other times manually reported by children or their parents (108).

### CG treatment

The CG treatment is another determinant of observed effectiveness for SC and MVPA. Interventions tend to demonstrate stronger effects when compared against inactive or non-digital control conditions, whereas comparisons with alternative digital interventions often yield attenuated or non-significant results, as reported in previous studies (96, 109). For example, gamified interventions had greater effects when compared with inactive control groups than when compared with active control groups receiving non-gamified interventions (96). When interventions are compared to inactive or non-digital control conditions (e.g., wait-list, usual care), larger effect sizes are typically observed because these comparators do not provide any structured stimulus to change behavior (110, 111). In such cases, the contrast between groups reflects both the specific effect of the intervention and the absence of any competing behavioral influence in the control arm. In contrast, when interventions are evaluated against active or alternative digital control conditions, the difference between groups is often reduced (112). This is because both arms may include behaviorally relevant components such as self-monitoring, feedback, or goal setting, even if delivered through different platforms or with different intensity. As a result, the incremental benefit of the intervention under study becomes smaller and may not reach statistical significance, not necessarily due to a lack of efficacy, but rather due to a “dilution” of effects caused by an active comparator. This highlights the need for careful consideration of comparator conditions in future trials, as they can substantially influence the interpretation of intervention efficacy.

### Country, age, weight status, setting, follow-up duration

Other factors influencing intervention effectiveness include the country of origin of the study, participant age and weight status, setting, and follow-up duration.

Regarding the geographic origin, interventions conducted in Europe were found to be ineffective in increasing PA outcomes. This may be due to the high heterogeneity among European studies (e.g., variations in follow-up durations were observed). Therefore, future European studies should strive for greater methodological consistency, which would reduce heterogeneity and enable more reliable comparisons.

Another key finding is that the age of the target population is a moderator of intervention effectiveness. Interventions targeting younger children appeared to be more successful in increasing step counts, whereas those focusing on older students showed greater improvements in MVPA. Although there is no clear evidence in the literature, this likely reflects developmental differences in activity patterns. Younger children engage primarily in unstructured, ambulatory activity, making total movement more responsive to change, with a consequent simple accumulation of movement that easily influences step counts. In contrast, older youth participate more in structured and intensity-based activities (sports, training) that are more likely to affect MVPA rather than total steps (113). Overall, these findings suggest the need to tailor interventions according to developmental stage, emphasizing movement volume in younger children and activity intensity in older populations.

Another key question is why digital interventions have generally been ineffective in overweight or obese children. Subgroup analyses did not reveal significant differences by weight status, except when a mix of digital devices was used, which proved more effective. Although these findings are based on only two observations from the same study (69), they suggest that more targeted, condition-specific interventions may be required for overweight or obese children.

Simple interventions involving active video games or pedometers may be insufficient for this population. Instead, multidisciplinary approaches are recommended that combine digital tools with other behavior change strategies such as nutritional guidance or alternative leisure-time physical activities. Researchers should take this into account when designing interventions for children with overweight or obesity.

With regard to the setting of the intervention, this MA found that school-based RCTs were not effective overall, in contrast to those conducted in home environments. A plausible explanation is that home contexts may offer greater flexibility, autonomy, and family support, all of which can facilitate sustained engagement in PA. In contrast, school-based interventions may be constrained by structural factors such as limited time, competing academic priorities, and reduced individualization (114). These findings have important implications for both policy and school-based interventions. The limited effectiveness of digital interventions in school settings suggests that their impact may be constrained by structural factors such as limited autonomy, time restrictions, or competing curricular demands (115, 116). In contrast, their greater effectiveness in home environments indicates that digital tools may be better suited to contexts where children and adolescents can engage more flexibly and self-direct their activity (117). Therefore, public health strategies should consider prioritizing the use of digital interventions in out-of-school settings, while in school, they may need to be complemented by structured, supervised, and context-specific approaches to effectively promote PA.

The duration of the intervention also appears to play a critical role. Medium-term interventions showed greater effectiveness compared to both short- and long-term programs. In the short term, interventions may be insufficient to establish stable behavioral habits (118), while in the long term, the novelty and appeal of digital tools may diminish, leading to reduced engagement (119). These findings suggest that future interventions should carefully balance duration and intensity, potentially incorporating strategies to maintain user interest over time. The ultimate goal should be to encourage young people to engage in free-time activities that combine digital engagement with physical movement, fostering an active lifestyle throughout childhood, adolescence, and ideally into adulthood.

### Strengths

One major strength of this meta-analysis is the inclusion of detailed sensitivity and subgroup analyses, which provide deeper insights into how different factors influence the effectiveness of digital tools in promoting physical activity. This adds originality and practical value to the study. Furthermore, the inclusion of PIs alongside CIs is rare in meta-analyses (120, 121).

While the CI reflects the precision of the mean effect, the PI estimates the range within which the effect of a future study may fall. This distinction can help researchers and policymakers better plan future interventions and design more effective strategies to promote physical activity in youth.

### Limitations

One limitation of this MA is the lack of gender-specific data in the extracted RCTs, preventing analysis by sex. Future studies should aim to report outcomes by gender, as intervention effects may differ between males and females.

A small study effect was only detected in RCTs evaluating MVPA as the outcome. This suggests a potential publication bias due to a lack of small studies favoring the control group, possibly inflating the estimated MVPA effect by 0.85 min/day. As a result, these findings should be interpreted with caution.

## Conclusions

Overall, these findings suggest that digital interventions can be effective tools for promoting physical activity in young populations, particularly when they are designed as multifaceted, context-sensitive programs that integrate technological and human elements. Future research should aim to disentangle the relative contributions of these components, optimize intervention design for different population subgroups, and explore strategies to sustain long-term engagement and effectiveness.

## Data Availability

The raw data supporting the conclusions of this article will be made available by the authors, without undue reservation.

## References

[1] Poitras VJ Gray CE Borghese MM Carson V Chaput JP Janssen I. Systematic review of the relationships between objectively measured physical activity and health indicators in school-aged children and youth. Appl Physiol Nutr Metab. (2016) 41(Suppl. 3):S197–239. 10.1139/apnm-2015-0663 27306431

[2] Guthold R Stevens GA Riley LM Bull FC. Global trends in insufficient physical activity among adolescents: a pooled analysis of 298 population-based surveys with 1·6 million participants. Lancet Child Adolesc Health. (2020) 4(1):23–35. 10.1016/S2352-4642(19)30323-2 31761562 PMC6919336

[3] Farooq A Martin A Janssen X Wilson MG Gibson A Hughes A. Longitudinal changes in moderate-to-vigorous-intensity physical activity in children and adolescents: a systematic review and meta-analysis. Obes Rev. (2020) 21(1):e12953. 10.1111/obr.12953 31646739 PMC6916562

[4] Tremblay MS Barnes AS Saunders JD Carson TJ Latimer-Cheung V Altenbarg TM. Sedentary behavior research network (SBRN) – terminology consensus project process and outcome. Int J Behav Nutr Phys Act. (2017) 14(1):75. 10.1186/s12966-017-0525-8 28599680 PMC5466781

[5] WHO. Guidelines on Physical Activity and Sedentary Behaviour. 1st ed. Geneva: World Health Organization (2020). 1 33369898

[6] Kraus WE Janz KF Powell KE Campbell WW Jakicic JM Troiano RP. Daily step counts for measuring physical activity exposure and its relation to health. Med Sci Sports Exerc. (2019) 51(6):1206–12. 10.1249/MSS.0000000000001932 31095077 PMC6527133

[7] Hall KS Hyde ET Bassett DR Carlson SA Carnethon MR Ekelund U. Systematic review of the prospective association of daily step counts with risk of mortality, cardiovascular disease, and dysglycemia. Int J Behav Nutr Phys Act. (2020) 17(1):78. 10.1186/s12966-020-00978-9 32563261 PMC7305604

[8] Stojanović S Andrieieva O Trajković N. Associations between number of steps and health outcomes in children and adolescents: a systematic review and meta-analysis. BMC Public Health. (2024) 24(1):3310. 10.1186/s12889-024-20835-9 39604923 PMC11603645

[9] Baumann H Fiedler J Wunsch K Woll A Wollesen B. mHealth interventions to reduce physical inactivity and sedentary behavior in children and adolescents: systematic review and meta-analysis of randomized controlled trials. JMIR mHealth uHealth. (2022) 10(5):e35920. 10.2196/35920 35544294 PMC9133983

[10] Emberson MA Lalande A Wang D McDonough DJ Liu W Gao Z. Effectiveness of smartphone-based physical activity interventions on Individuals’ health outcomes: a systematic review. BioMed Res Int. (2021) 2021:1–13. 10.1155/2021/6296896 34409104 PMC8367594

[11] He Z Wu H Yu F Fu J Sun S Huang T. Effects of smartphone-based interventions on physical activity in children and adolescents: systematic review and meta-analysis. JMIR mHealth uHealth. (2021) 9(2):e22601. 10.2196/22601 33522980 PMC7884215

[12] Tudor-Locke C Craig CL Beets MW Belton S Cardon GM Duncan S. How many steps/day are enough? For children and adolescents. Int J Behav Nutr Phys Act. (2011) 8(1):78. 10.1186/1479-5868-8-78 21798014 PMC3166269

[13] Da Silva MP Fontana FE Callahan E Mazzardo O De Campos W. Step-count guidelines for children and adolescents: a systematic review. J Phys Act Health. (2015) 12(8):1184–91. 10.1123/jpah.2014-0202 25271673

[14] Colley RC Janssen I Tremblay MS. Daily step target to measure adherence to physical activity guidelines in children. Med Sci Sports Exerc. (2012) 44(5):977–82. 10.1249/MSS.0b013e31823f23b1 22051570

[15] Fan RS Jiang JJ Zhou QY Zhang XY Wu ZH Ji L. Digital health interventions to promote physical activity among adolescents: systematic review. J Med Internet Res. (2026) 28:e82395. 10.2196/82395 41759087 PMC13148130

[16] Uijtdewilligen L Nauta J Singh AS Van Mechelen W Twisk JWR Van Der Horst K. Determinants of physical activity and sedentary behaviour in young people: a review and quality synthesis of prospective studies. Br J Sports Med. (2011) 45(11):896–905. 10.1136/bjsports-2011-090197 21836173

[17] Craggs C Corder K Van Sluijs EMF Griffin SJ. Determinants of change in physical activity in children and adolescents. Am J Prev Med. (2011) 40(6):645–58. 10.1016/j.amepre.2011.02.025 21565658 PMC3100507

[18] Hu D Zhou S Crowley-McHattan ZJ Liu Z. Factors that influence participation in physical activity in school-aged children and adolescents: a systematic review from the social ecological model perspective. Int J Environ Res Public Health. (2021) 18(6):3147. 10.3390/ijerph18063147 33803733 PMC8003258

[19] Cai J Zhao Y Wang J Wang L. Influencing factors of children’s physical activity in family. BMC Public Health. (2022) 22(1):787. 10.1186/s12889-022-13235-4 35440083 PMC9020037

[20] World Health Organization. WHO Guideline: Recommendations on Digital Interventions for Health System Strengthening. Geneva: World Health Organization (2019). Available online at: https://iris.who.int/handle/10665/311941 (Accessed January 22, 2025). 31162915

[21] De Santis KK Jahnel T Matthias K Mergenthal L Al Khayyal H Zeeb H. Evaluation of digital interventions for physical activity promotion: scoping review. JMIR Public Health Surveill. (2022) 8(5):e37820. 10.2196/37820 35604757 PMC9171604

[22] McCallum C Rooksby J Gray CM. Evaluating the impact of physical activity apps and wearables: interdisciplinary review. JMIR mHealth uHealth. (2018) 6(3):e58. 10.2196/mhealth.9054 29572200 PMC5889496

[23] Tripicchio GL Jones GJ Hart CN Hyun M DeSabato E Giddings A. A digitally enhanced home-based physical activity intervention for high-risk middle school youth during COVID-19. Transl Behav Med. (2023) 13(1):17–24. 10.1093/tbm/ibab151 34850218 PMC8690196

[24] Van Sluijs EMF Ekelund U Crochemore-Silva I Guthold R Ha A Lubans D. Physical activity behaviours in adolescence: current evidence and opportunities for intervention. Lancet. (2021) 398(10298):429–42. 10.1016/S0140-6736(21)01259-9 34302767 PMC7612669

[25] Kracht CL Hutchesson M Ahmed M Müller AM Ashton LM Brown HM. E-&mHealth interventions targeting nutrition, physical activity, sedentary behavior, and/or obesity among children: a scoping review of systematic reviews and meta-analyses. Obes Rev. (2021) 22(12):e13331. 10.1111/obr.13331 34476890 PMC8865754

[26] Kang HS Exworthy M. Wearing the future—wearables to empower users to take greater responsibility for their health and care: scoping review. JMIR mHealth uHealth. (2022) 10(7):e35684. 10.2196/35684 35830222 PMC9330198

[27] Robertson W Stewart-Brown S Wilcock E Oldfield M Thorogood M. Utility of accelerometers to measure physical activity in children attending an obesity treatment intervention. J Obes. (2011) 2011:1–8. 10.1155/2011/398918 PMC2952817 20953356

[28] Thompson WR. Worldwide survey of fitness trends for 2020. ACSMS Health Fit J. (2019) 23(6):10–8. 10.1249/FIT.0000000000000526

[29] Grady A Pearson N Lamont H Leigh L Wolfenden L Barnes C. The effectiveness of strategies to improve user engagement with digital health interventions targeting nutrition, physical activity, and overweight and obesity: systematic review and meta-analysis. J Med Internet Res. (2023) 25:e47987. 10.2196/47987 38113062 PMC10762625

[30] Tabacchi G Scardina A Amato A Giardina M Accardi G Di Liberto V. Digital tools’ effectiveness on physical activity outcomes in children and adolescents: umbrella review. JMIR Public Health Surveill. (2026) 12:e75769. 10.2196/75769 41877408 PMC13013097

[31] Rethlefsen ML Kirtley S Waffenschmidt S Ayala AP Moher D Page MJ. PRISMA-S: an extension to the PRISMA statement for reporting literature searches in systematic reviews. Syst Rev. (2021) 10(1):39. 10.1186/s13643-020-01542-z 33499930 PMC7839230

[32] Müller AM Maher CA Vandelanotte C Hingle M Middelweerd A Lopez ML. Physical activity, sedentary behavior, and diet-related eHealth and mHealth research: bibliometric analysis. J Med Internet Res. (2018) 20(4):e122. 10.2196/jmir.8954 29669703 PMC5932335

[33] Allafi AR. Effects of rewards and pedometer-feedback on children’s physical activity: a school-based intervention study. Prog Nutr. (2020) 22(1):122–6. 10.23751/pn.v22i1.8117

[34] Baldursdottir B Taehtinen RE Sigfusdottir ID Krettek A Valdimarsdottir HB. Impact of a physical activity intervention on adolescents’ subjective sleep quality: a pilot study. Glob Health Promot. (2017) 24(4):14–22. 10.1177/1757975915626112 27173502

[35] Baranowski T Baranowski J Thompson D Buday R Jago R Griffith MJ. Video game play, child diet, and physical activity behavior change. Am J Prev Med. (2011) 40(1):33–8. 10.1016/j.amepre.2010.09.029 21146765 PMC3032382

[36] Baranowski T Abdelsamad D Baranowski J O’Connor TM Thompson D Barnett A. Impact of an active video game on healthy children’s physical activity. Pediatrics. (2012) 129(3):e636–42. 10.1542/peds.2011-2050 22371457 PMC3289528

[37] Baranowski T Baranowski J Chen TA Buday R Beltran A Dadabhoy H. Videogames that encourage healthy behavior did not alter fasting insulin or other diabetes risks in children: randomized clinical trial. Games Health J. (2019) 8(4):257–64. 10.1089/g4h.2018.0097 30964335 PMC6686687

[38] Brannon EE Cushing CC Walters RW Crick C Noser AE Mullins LL. Goal feedback from whom? A physical activity intervention using an N-of-1 RCT. Psychol Health. (2018) 33(6):701–12. 10.1080/08870446.2017.1385783 28988493

[39] Dewar DL Morgan PJ Plotnikoff RC Okely AD Collins CE Batterham M. The nutrition and enjoyable activity for teen girls study. Am J Prev Med. (2013) 45(3):313–7. 10.1016/j.amepre.2013.04.014 23953358

[40] Dewar DL Morgan PJ Plotnikoff RC Okely AD Batterham M Lubans DR. Exploring changes in physical activity, sedentary behaviors and hypothesized mediators in the NEAT girls group randomized controlled trial. J Sci Med Sport. (2014) 17(1):39–46. 10.1016/j.jsams.2013.02.003 23506657

[41] Direito A Jiang Y Whittaker R Maddison R. Apps for IMproving FITness and increasing physical activity among young people: the AIMFIT pragmatic randomized controlled trial. J Med Internet Res. (2015) 17(8):e210. 10.2196/jmir.4568 26316499 PMC4642788

[42] Duncan M Staples V. The impact of a school-based active video game play intervention on children’s physical activity during recess. Hum Mov. (2010) 11(1):95–99. 10.2478/v10038-009-0023-1

[43] Errickson SP Maloney AE Thorpe D Giuliani C Rosenberg AM. “Dance Dance Revolution” used by 7- and 8-year-olds to boost physical activity: is coaching necessary for adherence to an exercise prescription? Games Health J. (2012) 1(1):45–50. 10.1089/g4h.2011.0028 26196431

[44] Ezendam NPM. Evaluation of the web-based computer-tailored FATaintPHAT intervention to promote energy balance among adolescents: results from a school cluster randomized trial. Arch Pediatr Adolesc Med. (2012) 166(3):248. 10.1001/archpediatrics.2011.204 22064878

[45] Garde A Umedaly A Abulnaga SM Robertson L Junker A Chanoine JP. Assessment of a mobile game (“MobileKids Monster Manor”) to promote physical activity among children. Games Health J. (2015) 4(2):149–58. 10.1089/g4h.2014.0095 26181809

[46] Garde A Umedaly A Abulnaga SM Junker A Chanoine JP Johnson M. Evaluation of a novel mobile exergame in a school-based environment. Cyberpsychology Behav Soc Netw. (2016) 19(3):186–92. 10.1089/cyber.2015.0281 26882222

[47] Garde A Chowdhury M Rollinson AU Johnson M Prescod P Chanoine JP. A multi-week assessment of a mobile exergame intervention in an elementary school. Games Health J. (2018) 7(1):43–50. 10.1089/g4h.2017.0023 29394109

[48] Graves LEF Ridgers ND Atkinson G Stratton G. The effect of active video gaming on children’s physical activity, behavior preferences and body composition. Pediatr Exerc Sci. (2010) 22(4):535–46. 10.1123/pes.22.4.535 21242603

[49] Guagliano JM Armitage SM Brown HE Coombes E Fusco F Hughes C. A whole family-based physical activity promotion intervention: findings from the families reporting every step to health (FRESH) pilot randomised controlled trial. Int J Behav Nutr Phys Act. (2020) 17(1):120. 10.1186/s12966-020-01025-3 32962724 PMC7510101

[50] Guthrie N Bradlyn A Thompson SK Yen S Haritatos J Dillon F. Development of an accelerometer-linked online intervention system to promote physical activity in adolescents. PLoS One. (2015) 10(5):e0128639. 10.1371/journal.pone.0128639 26010359 PMC4444279

[51] Jake-Schoffman DE Turner-McGrievy G Wilcox S Moore JB Hussey JR Kaczynski AT. The mFIT (motivating families with interactive technology) study: a randomized pilot to promote physical activity and healthy eating through mobile technology. J Technol Behav Sci. (2018) 3(3):179–89. 10.1007/s41347-018-0052-8

[52] Jauho AM Pyky R Ahola R Kangas M Virtanen P Korpelainen R. Effect of wrist-worn activity monitor feedback on physical activity behavior: a randomized controlled trial in Finnish young men. Prev Med Rep. (2015) 2:628–34. 10.1016/j.pmedr.2015.07.005 26844128 PMC4721342

[53] Lau PWC Wang JJ Maddison R. A randomized-controlled trial of school-based active videogame intervention on Chinese children’s aerobic fitness, physical activity level, and psychological correlates. Games Health J. (2016) 5(6):405–12. 10.1089/g4h.2016.0057 27855265

[54] Leinonen AM Pyky R Ahola R Kangas M Siirtola P Luoto T. Feasibility of gamified mobile service aimed at physical activation in young men: population-based randomized controlled study (MOPO). JMIR mHealth uHealth. (2017) 5(10):e146. 10.2196/mhealth.6675 29017991 PMC5654732

[55] Lubans DR. Preventing obesity among adolescent girls: one-year outcomes of the nutrition and enjoyable activity for teen girls (NEAT girls) cluster randomized controlled trial. Arch Pediatr Adolesc Med. (2012) 166(9):821. 10.1001/archpediatrics.2012.41 22566517

[56] Lubans DR Smith JJ Plotnikoff RC Dally KA Okely AD Salmon J. Assessing the sustained impact of a school-based obesity prevention program for adolescent boys: the ATLAS cluster randomized controlled trial. Int J Behav Nutr Phys Act. (2016) 13(1):92. 10.1186/s12966-016-0420-8 27542825 PMC4992277

[57] Mack I Reiband N Etges C Eichhorn S Schaeffeler N Zurstiege G. The kids obesity prevention program: cluster randomized controlled trial to evaluate a serious game for the prevention and treatment of childhood obesity. J Med Internet Res. (2020) 22(4):e15725. 10.2196/15725 32329742 PMC7210499

[58] Maddison R Foley L Ni Mhurchu C Jiang Y Jull A Prapavessis H. Effects of active video games on body composition: a randomized controlled trial. Am J Clin Nutr. (2011) 94(1):156–63. 10.3945/ajcn.110.009142 21562081

[59] Maddison R Marsh S Foley L Epstein LH Olds T Dewes O. Screen-time weight-loss intervention targeting children at home (SWITCH): a randomized controlled trial. Int J Behav Nutr Phys Act. (2014) 11(1):111. 10.1186/s12966-014-0111-2 25204320 PMC4174282

[60] Maloney AE Threlkeld KA Cook WL. Comparative effectiveness of a 12-week physical activity intervention for overweight and obese youth: exergaming with “Dance Dance Revolution”. Games Health J. (2012) 1(2):96–103. 10.1089/g4h.2011.0009 26193183

[61] Morgan PJ Young MD Barnes AT Eather N Pollock ER Lubans DR. Engaging fathers to increase physical activity in girls: the “dads and daughters exercising and empowered” (DADEE) randomized controlled trial. Ann Behav Med. (2019) 53(1):39–52. 10.1093/abm/kay015 29648571

[62] Morris JL Daly-Smith A Defeyter MA McKenna J Zwolinsky S Lloyd S. A pedometer-based physically active learning intervention: the importance of using preintervention physical activity categories to assess effectiveness. Pediatr Exerc Sci. (2019) 31(3):356–62. 10.1123/pes.2018-0128 30612529

[63] Ngo CS Pan C Finkelstein EA Lee C Wong IB Ong J. A cluster randomised controlled trial evaluating an incentive-based outdoor physical activity programme to increase outdoor time and prevent myopia in children. Ophthalmic Physiol Opt. (2014) 34(3):362–8. 10.1111/opo.12112 24460536

[64] Pfeiffer KA Robbins LB Ling J Sharma DB Dalimonte-Merckling DM Voskuil VR. Effects of the girls on the move randomized trial on adiposity and aerobic performance (secondary outcomes) in low-income adolescent girls. Pediatr Obes. (2019) 14(11):e12559. 10.1111/ijpo.12559 31267695 PMC6982403

[65] Pope L Garnett B Dibble M. Lessons learned through the implementation of an eHealth physical activity gaming intervention with high school youth. Games Health J. (2018) 7(2):136–42. 10.1089/g4h.2017.0164 29393679

[66] Ridgers ND Timperio A Ball K Lai SK Brown H Macfarlane S. Effect of commercial wearables and digital behaviour change resources on the physical activity of adolescents attending schools in socio-economically disadvantaged areas: the RAW-PA cluster-randomised controlled trial. Int J Behav Nutr Phys Act. (2021) 18(1):52. 10.1186/s12966-021-01110-1 33845853 PMC8042874

[67] Robbins LB Ling J Sharma DB Dalimonte-Merckling DM Voskuil VR Resnicow K. Intervention effects of “girls on the move” on increasing physical activity: a group randomized trial. Ann Behav Med. (2019) 53(5):493–500. 10.1093/abm/kay054 29985968 PMC6322965

[68] Robertson J Macvean A Fawkner S Baker G Jepson RG. Savouring our mistakes: learning from the FitQuest project. Int J Child-Comput Interact. (2018) 16:55–67. 10.1016/j.ijcci.2017.12.003

[69] Ruotsalainen H Kyngäs H Tammelin T Heikkinen H Kääriäinen M. Effectiveness of Facebook-delivered lifestyle counselling and physical activity self-monitoring on physical activity and body mass index in overweight and obese adolescents: a randomized controlled trial. Nurs Res Pract. (2015) 2015:1–14. 10.1155/2015/159205 PMC4678089 26697218

[70] Seah MLC Koh KT. The efficacy of using mobile applications in changing adolescent girls’ physical activity behaviour during weekends. Eur Phys Educ Rev. (2021) 27(1):113–31. 10.1177/1356336X20930741

[71] Smith JJ Morgan PJ Plotnikoff RC Dally KA Salmon J Okely AD. Smart-phone obesity prevention trial for adolescent boys in low-income communities: the ATLAS RCT. Pediatrics. (2014) 134(3):e723–31. 10.1542/peds.2014-1012 25157000

[72] Staiano AE Beyl RA Hsia DS Katzmarzyk PT Newton RL. Twelve weeks of dance exergaming in overweight and obese adolescent girls: transfer effects on physical activity, screen time, and self-efficacy. J Sport Health Sci. (2017) 6(1):4–10. 10.1016/j.jshs.2016.11.005 28491483 PMC5421642

[73] Staiano AE Beyl RA Guan W Hendrick CA Hsia DS Newton RL. Home-based exergaming among children with overweight and obesity: a randomized clinical trial. Pediatr Obes. (2018) 13(11):724–33. 10.1111/ijpo.12438 30027607 PMC6203598

[74] Thompson D Cantu D Ramirez B Cullen KW Baranowski T Mendoza J. Texting to increase adolescent physical activity: feasibility assessment. Am J Health Behav. (2016) 40(4):472–83. 10.5993/AJHB.40.4.9 27338994 PMC4922515

[75] Trost SG Sundal D Foster GD Lent MR Vojta D. Effects of a pediatric weight management program with and without active video games: a randomized trial. JAMA Pediatr. (2014) 168(5):407. 10.1001/jamapediatrics.2013.3436 24589566

[76] Van Woudenberg TJ Bevelander KE Burk WJ Smit CR Buijs L Buijzen M. A randomized controlled trial testing a social network intervention to promote physical activity among adolescents. BMC Public Health. (2018) 18(1):542. 10.1186/s12889-018-5451-4 29685112 PMC5913789

[77] Van Woudenberg TJ Bevelander KE Burk WJ Smit CR Buijs L Buijzen M. Testing a social network intervention using vlogs to promote physical activity among adolescents: a randomized controlled trial. Front Psychol. (2020) 10:2913. 10.3389/fpsyg.2019.02913 31998181 PMC6967297

[78] DerSimonian R Laird N. Meta-analysis in clinical trials. Control Clin Trials. (1986) 7(3):177–88. 10.1016/0197-2456(86)90046-2 3802833

[79] Higgins JPT. Measuring inconsistency in meta-analyses. Br Med J. (2003) 327(7414):557–60. 10.1136/bmj.327.7414.557 12958120 PMC192859

[80] Deeks JJ Higgins JP Altman DG. Analysing data and undertaking meta-analyses. In: Higgins JP Green S, editors. Cochrane Handbook for Systematic Reviews of Interventions. 1st ed. Chichester: Wiley (2008). p. 243–96. 10.1002/9780470712184.ch9 (Accessed January 22, 2025).

[81] Riley RD Higgins JPT Deeks JJ. Interpretation of random effects meta-analyses. Br Med J. (2011) 342:d549. 10.1136/bmj.d549 21310794

[82] Ades AE Lu G Higgins JPT. The interpretation of random-effects meta-analysis in decision models. Med Decis Making. (2005) 25(6):646–54. 10.1177/0272989X05282643 16282215

[83] Sterne JAC Sutton AJ Ioannidis JPA Terrin N Jones DR Lau J. Recommendations for examining and interpreting funnel plot asymmetry in meta-analyses of randomised controlled trials. Br Med J. (2011) 343:d4002. 10.1136/bmj.d4002 21784880

[84] Egger M Smith GD Schneider M Minder C. Bias in meta-analysis detected by a simple, graphical test. Br Med J. (1997) 315(7109):629–34. 10.1136/bmj.315.7109.629 9310563 PMC2127453

[85] Duval S Tweedie R. Trim and fill: a simple funnel-plot–based method of testing and adjusting for publication bias in meta-analysis. Biometrics. (2000) 56(2):455–63. 10.1111/j.0006-341X.2000.00455.x 10877304

[86] Sterne JAC Savović J Page MJ Elbers RG Blencowe NS Boutron I. Rob 2: a revised tool for assessing risk of bias in randomised trials. Br Med J. (2019) 366:l4898. 10.1136/bmj.l4898 31462531

[87] Bandura A. Social Foundations of Thought and Action. In: The Health Psychology Reader. United Kingdom: SAGE Publications Ltd (2002). p. 94–106. 10.4135/9781446221129.n6 (Accessed September 9, 2024).

[88] Deci EL Ryan RM. Self-Determination Theory. In: Handbook of Theories of Social Psychology: Volume 1. United Kingdom: SAGE Publications Ltd (2012). p. 416–37. 10.4135/9781446249215.n21 (Accessed September 9, 2024).

[89] Prochaska JO Velicer WF. The transtheoretical model of health behavior change. Am J Health Promot. (1997) 12(1):38–48. 10.4278/0890-1171-12.1.38 10170434

[90] Longobucco Y Ricci M Scrimaglia S Camedda C Dallolio L Masini A. Effects of school nurse-led interventions in collaboration with kinesiologists in promoting physical activity and reducing sedentary behaviors in children and adolescents: a systematic review. Healthcare. (2023) 11(11):1567. 10.3390/healthcare11111567 37297707 PMC10252598

[91] Ahmadi MN Hamer M Gill JMR Murphy M Sanders JP Doherty A. Brief bouts of device-measured intermittent lifestyle physical activity and its association with major adverse cardiovascular events and mortality in people who do not exercise: a prospective cohort study. Lancet Public Health. (2023) 8(10):e800–10. 10.1016/S2468-2667(23)00183-4 37777289

[92] Chinapaw M Klakk H Møller NC Andersen LB Altenburg T Wedderkopp N. Total volume versus bouts: prospective relationship of physical activity and sedentary time with cardiometabolic risk in children. Int J Obes. (2018) 42(10):1733–42. 10.1038/s41366-018-0063-8 29717272

[93] Au WW Recchia F Fong DY Wong SHS Chan DKC Capio CM. Effect of wearable activity trackers on physical activity in children and adolescents: a systematic review and meta-analysis. Lancet Digit Health. (2024) 6(9):e625–39. 10.1016/S2589-7500(24)00139-0 39112110

[94] Creaser AV Clemes SA Costa S Hall J Ridgers ND Barber SE. The acceptability, feasibility, and effectiveness of wearable activity trackers for increasing physical activity in children and adolescents: a systematic review. Int J Environ Res Public Health. (2021) 18(12):6211. 10.3390/ijerph18126211 34201248 PMC8228417

[95] Mclaughlin M Delaney T Hall A Byaruhanga J Mackie P Grady A. Associations between digital health intervention engagement, physical activity, and sedentary behavior: systematic review and meta-analysis. J Med Internet Res. (2021) 23(2):e23180. 10.2196/23180 33605897 PMC8011420

[96] Mazeas A Duclos M Pereira B Chalabaev A. Evaluating the effectiveness of gamification on physical activity: systematic review and meta-analysis of randomized controlled trials. J Med Internet Res. (2022) 24(1):e26779. 10.2196/26779 34982715 PMC8767479

[97] Altmeyer M Schubhan M Krüger A Lessel P. A long-term investigation on the effects of (personalized) gamification on course participation in a gym. arXiv Preprint ArXiv. (2021). 10.48550/ARXIV.2107.12597

[98] Wang M Xu J Zhou X Li X Zheng Y. Effectiveness of gamification interventions to improve physical activity and sedentary behavior in children and adolescents: systematic review and meta-analysis. JMIR Serious Games. (2025) 13:e68151. 10.2196/68151 40966596 PMC12445784

[99] Sal-de-Rellán A. Hernández-Suárez Á Hernaiz-Sánchez A. Gamification and motivation in adolescents. Systematic review from physical education. Front Psychol. (2025) 16:1575104. 10.3389/fpsyg.2025.1575104 40196203 PMC11973368

[100] Gkintoni E Vantaraki F Skoulidi C Anastassopoulos P Vantarakis A. Promoting physical and mental health among children and adolescents via gamification—a conceptual systematic review. Behav Sci. (2024) 14(2):102. 10.3390/bs14020102 38392455 PMC10886329

[101] Zhu J Chen M Jiang W Li J Liu X Jin H. Gamified digital exercise interventions for children and adolescents: protocol for a systematic review and behavior change technique analysis. Digit Health. (2025) 11:20552076251365017. 10.1177/20552076251365017 40761775 PMC12319270

[102] Xu L Shi H Shen M Ni Y Zhang X Pang Y. The effects of mHealth-based gamification interventions on participation in physical activity: systematic review. JMIR mHealth uHealth. (2022) 10(2):e27794. 10.2196/27794 35113034 PMC8855282

[103] Wang W Huang C Shen Y Cheng J Wang L. Effectiveness of step-count monitoring interventions in increasing physical activity among children and adolescents: a systematic review and meta-analysis. Digit Health. (2025) 11:20552076251374249. 10.1177/20552076251374249 40918079 PMC12409064

[104] Kim Y Lochbaum M. Comparison of polar active watch and waist- and wrist-worn ActiGraph accelerometers for measuring children’s physical activity levels during unstructured afterschool programs. Int J Environ Res Public Health. (2018) 15(10):2268. 10.3390/ijerph15102268 30332785 PMC6209975

[105] Ferguson T Rowlands AV Olds T Maher C. The validity of consumer-level, activity monitors in healthy adults worn in free-living conditions: a cross-sectional study. Int J Behav Nutr Phys Act. (2015) 12(1):42. 10.1186/s12966-015-0201-9 25890168 PMC4416251

[106] Rognmo K Opdal IM Handegård BH Horsch A Lillevoll K Furberg AS. The relationship between self-reported and device-based measurements of physical activity and mental distress among adolescents: results from the fit futures study. BMC Public Health. (2025) 25(1):2617. 10.1186/s12889-025-23902-x 40753245 PMC12317580

[107] Zhang W Xiong K Zhu C Evans R Zhou L Podrini C. Promoting child and adolescent health through wearable technology: a systematic review. Digit Health. (2024) 10:20552076241260507. 10.1177/20552076241260507 38868368 PMC11168039

[108] Smith L Michels LV Jat A Faulkner J Keast J Dambha-Miller H. Wearable device-based measurement of physical activity in populations at risk of health inequity: a scoping review. (2026). arXiv [Preprint]. 10.20944/preprints202601.0667.v1 (Accessed April 20, 2026)

[109] Fichtner UA Tinsel I Sehlbrede M Maiwald P Bischoff M Metzner G. Effects of a digital intervention on physical activity in adults: a randomized controlled trial in a large-scale sample. Internet Interv. (2024) 37:100762. 10.1016/j.invent.2024.100762 39211309 PMC11359763

[110] Caldwell DM Palmer JC Webster KE Davies SR Hughes H Rona J. Exploring the moderating effect of control group type on intervention effectiveness in school-based anxiety and depression prevention: findings from a rapid review and network meta-analysis. Prev Sci. (2025) 26(2):175–92. 10.1007/s11121-025-01786-y 39937398 PMC11891107

[111] Lane AM Beedie CJ Devonport TJ Friesen AP. Considerations of control groups: comparing active-control with no treatment for examining the effects of brief intervention. Sports. (2021) 9(11):156. 10.3390/sports9110156 34822355 PMC8623878

[112] Au J Gibson BC Bunarjo K Buschkuehl M Jaeggi SM. Quantifying the difference between active and passive control groups in cognitive interventions using two meta-analytical approaches. J Cogn Enhanc. (2020) 4(2):192–210. 10.1007/s41465-020-00164-6 34337311 PMC8320766

[113] Atkin AJ Sherar LB Ekelund U Hansen BH Andersen LB Anderssen S. Age-related change in children’s physical activity and sedentary time: the International Children’s Accelerometry Database (ICAD). PLoS One. (2025) 20(9):e0327394. 10.1371/journal.pone.0327394 40929102 PMC12422510

[114] Navarra GA Thomas E Scardina A Izadi M Zangla D De Dominicis S. Effective strategies for promoting physical activity through the use of digital media among school-age children: a systematic review. Sustainability. (2021) 13(20):11270. 10.3390/su132011270

[115] Cassar S Salmon J Timperio A Naylor PJ Van Nassau F Contardo Ayala AM. Adoption, implementation and sustainability of school-based physical activity and sedentary behaviour interventions in real-world settings: a systematic review. Int J Behav Nutr Phys Act. (2019) 16(1):120. 10.1186/s12966-019-0876-4 31791341 PMC6889569

[116] Ha T Moon J Yu H Fan X Paulson L. A systematic review of technology-infused physical activity interventions in K-12 school settings: effectiveness, roles, and implementation strategies. Int J Behav Nutr Phys Act. (2025) 22(1):113. 10.1186/s12966-025-01811-x 40849486 PMC12374309

[117] Seims AL Hall J Bingham DD Creaser A Christoforou A Barber S. Interventions targeting children and young people’s physical activity behavior at home: a systematic review. PLoS One. (2023) 18(8):e0289831. 10.1371/journal.pone.0289831 37556477 PMC10411747

[118] Wu J Xu Z Liu H Chen X Huang L Shi Q. Effects of commercial exergames and conventional exercises on improving executive functions in children and adolescents: meta-analysis of randomized controlled trials. JMIR Serious Games. (2023) 11:e42697. 10.2196/42697 37856191 PMC10623224

[119] Gulati AK Lobo RE Bhat V Bora N Sinha MK. Young adults journey with digital fitness tools-A qualitative study on use of fitness tracking device. F1000Res. (2024) 13:1296. 10.12688/f1000research.158037.1 39534658 PMC11555328

[120] Borg DN Impellizzeri FM Borg SJ Hutchins KP Stewart IB Jones T. Meta-analysis prediction intervals are under reported in sport and exercise medicine. Scand J Med Sci Sports. (2024) 34(3):e14603. 10.1111/sms.14603 38501202

[121] Sainani KL Borg DN Caldwell AR Butson ML Tenan MS Vickers AJ. Call to increase statistical collaboration in sports science, sport and exercise medicine and sports physiotherapy. Br J Sports Med. (2021) 55(2):118–22. 10.1136/bjsports-2020-102607 32816788 PMC7788220

