# Supplementary material for: Meta-analyses of randomized controlled trials assessing the effect of digital tools on step count and moderate-to-vigorous physical activity in healthy children and adolescents
Source: Front Digit Health. 2026 Jun 4;8:1701301. doi: 10.3389/fdgth.2026.1701301 (PMC13275700; doi:10.3389/fdgth.2026.1701301)
Supplement: Supplementary file 1 [file Supplementaryfile1.docx]

**Supplementary Material 1. Characteristics of the retrieved RCTs.**

| **First author, year, country** | **Intervention name** | **Focus task** | **Theoretical foundation** | **Setting** | **Age, school attendance (special population)** | **Sample dimension (M, F)** | **Follow-up** |
| --- | --- | --- | --- | --- | --- | --- | --- |
| Allafi, 2020, Kuwait | NA | PA | NA | School | 9-11, Elementary + Middle | 225 tot (110M, 115F): IG1 85; IG2 68; CG 72 | 50 minutes |
| Baldursdóttir, 2017, Iceland | NA | Sleep | NA | School | 15-16, High | 53 tot (21M, 32F): IG 26 (12M, 14F); CG 27 (9M, 18F) | 3 weeks |
| Baranowski, 2011, USA | Diab and Nano study | PA, diet | NA | Home, primary care | 10-12, Elementary + Middle (normal and overweight) | 153 tot: IG 103 (58M, 45F); CG 50 (28M, 22F) | immediately after Nano (P2), 2 months |
| Baranowski, 2012, USA | NA | PA | NA | Home, primary care | 9-12, Elementary + Middle (normal and overweight) | 78 tot (40M, 38F): IG 4; CG 37 | 1 week, 6 weeks, 7 weeks, 12 weeks |
| Baranowski, 2019, USA | Diab and Nano study | Health | NA | Home, primary care | 10–12, Elementary + Middle (overweight/obese) | 145 tot (87M, 58F): IG 52; CG 93 | 3 months, 5 months |
| Direito, 2015, New Zealand | AIMFIT (Apps for IMproving FITness) intervention | PA, cardiorespiratory fitness | Self-Regulatory Behavior Change Techniques | Home | 14-17, High (insufficiently active) | 51 tot (22M, 29F): IG1 17 (8M, 9F); IG2 16 (6M, 10F); CG 18 (8M, 10F) | 8 weeks |
| Ezendam, 2012, Netherlands | The FATaintPHAT (VETisnietVET in Dutch) Web-based computer-tailored intervention | Energy balance | Theory of Planned Behavior, Precaution Adoption Process Model, implementation intentions | School | 12-13, Middle | 880 tot (482M, 398F): IG 485 (284M, 198F); CG 398 (198M, 200F) | 4 months, 24 months |
| Guagliano, 2020, UK | FRESH (Families Reporting Every Step to Health) intervention | PA | Self-Determination Theory | Home | 7-11, Elementary + Middle | 82 tot (49M, 33F): IG1 30 (15M, 15F); IG2 23 (19M, 4F); CG 29 (15M, 14F) | 8 weeks, 52 weeks |
| Jake-Schoffman, 2018, USA | mFIT (Motivating Families with Interactive Technology) study | PA, diet | Social Cognitive Theory, Theory of Planned Behavior | Home | 9-12, Elementary + Middle | 33 tot (12M, 21F): IG1 17 (9M, 8F); IG2 16 (4M, 12F) | 12 weeks |
| Lau, 2016, China | NA | PA, aerobic fitness, psychological aspects | NA | School | 8-11, Elementary | 80 tot (55M, 25F): IG 40 (29M, 11F); CG 40 (26M, 14F) | 12 weeks |
| Leinonen, 2017, Finland | MOPO study | PA | Transtheoretical Model of Behavior Change | Community/during the annual military call-ups | 16-20, High (boys) | 496 tot (only M): IG 250; CG 246 | 26 weeks |
| Lubans, 2012, Australia | NEAT Girls (Nutrition and Enjoyable Activity for Teen Girls) | Obesity | Social Ecological Model, incorporated policy and environmental changes | School | 12-14, Middle + High (girls) | 357 tot (only F): IG 178; CG 179 | 12 months |
| Maddison, 2011, New Zealand | NA | Body composition | NA | Community | 10-14, Middle (overweight/obese) | 322 tot (235M, 87F): IG 160 (116M, 44F); CG 162 (119M, 43F) | 12 weeks, 24 weeks |
| Morgan, 2019, Australia | DADEE (Dads And Daughters Exercising and Empowered) intervention | PA | NA | Home | 4-12, Elementary + Middle (girls) | 153 tot (only F): IG 74; CG 79 | 2 months, 9 months |
| Robertson, 2018, Scotland | NA | PA | Social Cognitive Theory | School | 10-11, Elementary | 215 tot (100M, 115F): IG 111 (55M, 56F), CG 104 (45M, 59F) | 5 weeks |
| Ruotsaleinen, 2015, Finland | NA | PA, weight | Theory of Compliance | Home | 13-16, High (overweight/obese) | 46 tot (14M, 32F): IG1 15 (5M, 10F); IG2 16 (5M, 11F); CG 15 (4M, 11F) | 12 weeks |
| Staiano, 2018, USA | GameSquad intervention | Adiposity, cardiometabolic health | Social Cognitive Theory | Home | 10-12, Elementary + Middle (overweigh/obese) | 46 tot (25M, 21F): IG 23; CG 23 | 24 weeks |
| Thompson, 2016, USA | NA | PA | Self Determination Theory | Community-based | 14-17, High | 160 tot (78M, 82F): IG1 40 (20M, 20F); IG2 40 (15M, 25F); IG3 40 (23M, 17F); CG 40 (20M, 20F) | 12 weeks |
| Trost, 2014, Australia | JOIN for ME program | PA, Weight | NA | Community | 8-12 (Mean 10.0, SD 1.7), Elementary + Middle | 75 tot (34M, 41F): IG 34 (15M, 19F); CG 41 (19M, 22F) | 16 weeks |

…continued

| **First author, year, country** | **Intervention arms** | **IG(s) and CG(s)** | **Digital component/s for intervention delivery (type, name)** | **Digital device for intervention delivery (model)** | **Non digital component/s of intervention** | **Outcome measure (tool)** |
| --- | --- | --- | --- | --- | --- | --- |
| Allafi, 2020, Kuwait | 3-arm | IG1 (=FB group) info on pedometer results; IG2 (=FB+R group) info on pedometers + incentive; CG use but no info on pedometers results | Wearable (pedometer) | Yamax Digiwalker SW-200 pedometer | Goals, incentives | Step count (Yamax Digiwalker SW-200 pedometer) |
| Baldursdóttir, 2017, Iceland | 2-arm | IG pedometers and step diaries; CG no pedometers and step diaries | Wearable (pedometer) | Yamax CW-701 pedometer | PA advices, step diary | Step count (Yamax CW-701 pedometer) |
| Baranowski, 2011, USA | 2-arm | IG played Diab and Nano in sequence; CG played diet and PA knowledge-based games on popular websites | Gamification (role-playing videogames - "Escape from Diab", "Nanoswarm: Invasion from Inner Space") | Computer/laptop | Incentives | MVPA (Actigraph AM-7164 accelerometer) |
| Baranowski, 2012, USA | 2-arm | IG active video games; CG inactive video game | Gamification (exergames for Nintendo Wii - "Active Life-Extreme Challenge", "EA Sports Active", "Dance Dance Revolution (DDR)", "Wii Fit Plus", "Wii Sports") | Console | Not used | MVPA (Actigraph GT3X accelerometer - elastic belt) |
| Baranowski, 2019, USA | 2-arm | IG played Diab and Nano; CG from waitlist | Gamification (role-playing videogames - "Escape from Diab", "Nanoswarm: Invasion from Inner Space") | Computer/laptop | Not used | MVPA (Actigraph GT3X accelerometer); gameplay data collected over the Internet |
| Direito, 2015, New Zealand | 3-arm | IG1 use of an immersive smartphone app; IG2 use of a non-immersive app; CG usual behavior | Smartphone apps (Immersive app: "Zombies Run! 5K training"; Nonimmersive app: "Get Run- ning-Couch to 5K") | Smartphone, iPod touch | Education, social networking/forums/messaging | MVPA (ActiGraph GT1M accelerometer) |
| Ezendam, 2012, Netherlands | 2-arm | IG have followed 8 modules behaviors related to energy balanc; CG no intervation; | Web-based (website) | Computer | Not used | Step count (Digiwalker SW200 YAMAX pedometer) |
| Guagliano, 2020, UK | 3-arm | IG1 FAM arm (family theory intervention online to increase PA + family action planners + FRESH website to choose weekly step challenge); IG2 PED arm (pedometer + info); CG no intervention | Web-based (FRESH website), wearable (pedometer) | Computer, Walk4Life pedometer | Not used | Step count (Walk4Life Pedometer), MVPA (ActiGraph GT3X accelerometer) |
| Jake-Schoffman, 2018, USA | 2-arm | IG1 (TECh+ family-based activities with mFIT website); IG2 (TECH invididual based activity); | mFIT website for Food and step logs, messaging function on mFIT website, app (TECH+ component) | Mobile phone | Program for activities; goals and rewards | Step count (ACCUSPLIT AX2720 pedometer), MVPA (Actigraph GT1M accelerometer) |
| Lau, 2016, China | 2-arm | IG played Xbox 360, twice/week during after-school hours, each for 60 min over 12 weeks in duration; CG no intervention | Gamification (exergame - Xbox 360 with Kinect sensor, and Depth camera) | Console | Not used | MVPA (ActiGraph GT3X+ accelerometer) |
| Leinonen, 2017, Finland | 2-arm | IG "MOPO group" wrist-worn physical activity monitor (Polar Active) with physical activity feedback and access to a gamified Web-based mobile service; CG no intervention | Gamification, Web (MOPOrtal), wearable (activity monitor) | Mobile phone, Polar Active activity monitor | Not used | MVPA (Polar Active activity monitor) |
| Lubans, 2012, Australia | 2-arm | IG following NEAT girls; CG non following NEAT girls | Text messaging, wearable (pedometer) | NA | School program (sport, interactive seminars, nutrition workshops, lunch-time PA sessions, PA and nutrition handbooks, parent newsletters) | MVPA (Actigraph MTI, 7164, GT1M, and GT3X accelerometer) |
| Maddison, 2011, New Zealand | 2-arm | IG Sony PlaStation upgrade package; CG normal video game play and no information received. | Gamification (exergame - Sony PlayStation EyeToy, a USB motion-capture camera to place a picture of the gamer on screen, which the gamer then interacts with - upgrade version: EyeToy camera, dance mat, a selection of active video games - Play3, Kinetic, Sport, and Dance Factory; Sony) | Console | Information about increasing PA, healthy eating, or weight loss; encourage to meet current PA recommendations | MVPA (Actigraph AM7164-2.2C accelerometer - right hip) |
| Morgan, 2019, Australia | 2-arm | IG DADEE intervention (app + pedometer); CG wait list | App (DADEE), wearable (pedometer) | Smartphone, Yamax SW200 pedometer | Group education and practical sessions, Daughters resources (tasks, folders), Sports skills program, Sport equipment pack | Step count (Yamax SW200 pedometer) |
| Robertson, 2018, Scotland | 2-arm | IG played the game (on Samsung Galaxy Ace II phones) during at least 1 h of PE lessons; CG standard mandated PE lessons. | Gamification (exergame - 8 exercise based mini-games for Android platform) | Smartphone | Not used | Step count, MVPA (New Lifestyles NL 1000 piezoelectric accelerometer) |
| Ruotsaleinen, 2015, Finland | 3-arm | IG1 FB-delivered lifestyle PA counselling + self-monitoring; IG2 FB-delivered lifestyle counselling; CG no FB, no self-monitoring. | Social media, wearable (accelerometer) | Computer/smartphone, Polar Active accelerometer | Not used | MVPA (Polar Active activity monitor) |
| Staiano, 2018, USA | 2-arm | IG GameSquad intervention; CG were asked to maintain their normal level of PA. | Gamification (exergame: Kinect®and Xbox 360®gaming console + a 24-week XboxLive subscription and four exergames (Your Shape:Fitness Evolved 2012,Just Dance 3,DisneylandAdventuresandKinect Sports Season 2), telehealth coaching (fitness coach over video chat in the exergame console) | Console | Booklet with instructions, and to compile for calculate compliance and adherence | Step count (Fitbit Zip), MVPA (ActiGraphGT3X+ accelerometer) |
| Thompson, 2016, USA | 4-arm | IG1 pedometer; IG2 pedometer + goal prompt; IG3 pedometer + goal prompt + theory-informed texts; CG no-treatment. | Text messaging, wearables (pedometers) | Smartphone, pedometer | Not used | Step count (New-Lifestyle AT-82), MVPA (Actigraph GT3X+ accelerometer) |
| Trost, 2014, Australia | 2-arm | IG program and active gaming; CG only program | Gamification (exergame - Xbox and Kinect + 2 active sports games Kinetic Adventures! and Kinect Sports) | Console | Pediatric weight management program (sessions on foods and drinks, reduction of screen time, goal setting, PA increase | MVPA (GT3X or GT3X+ ActiGraph accelerometer) |
