# Supplementary figures and images for: Meta-analyses of randomized controlled trials assessing the effect of digital tools on step count and moderate-to-vigorous physical activity in healthy children and adolescents
Source: Front Digit Health. 2026 Jun 4;8:1701301. doi: 10.3389/fdgth.2026.1701301 (PMC13275700; doi:10.3389/fdgth.2026.1701301)

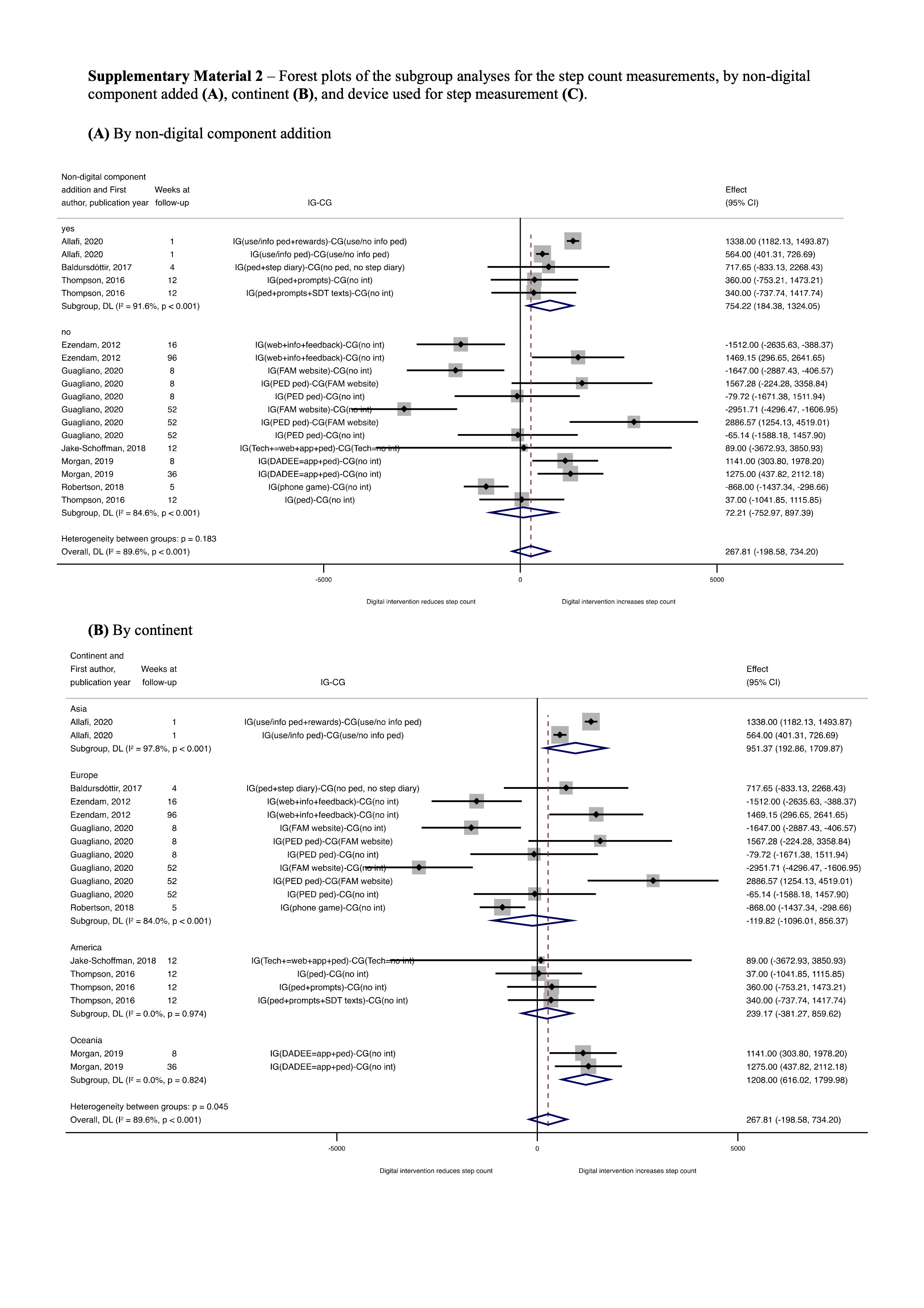

Supplement: Supplementary file 2 [file Supplementaryfile2.jpeg]

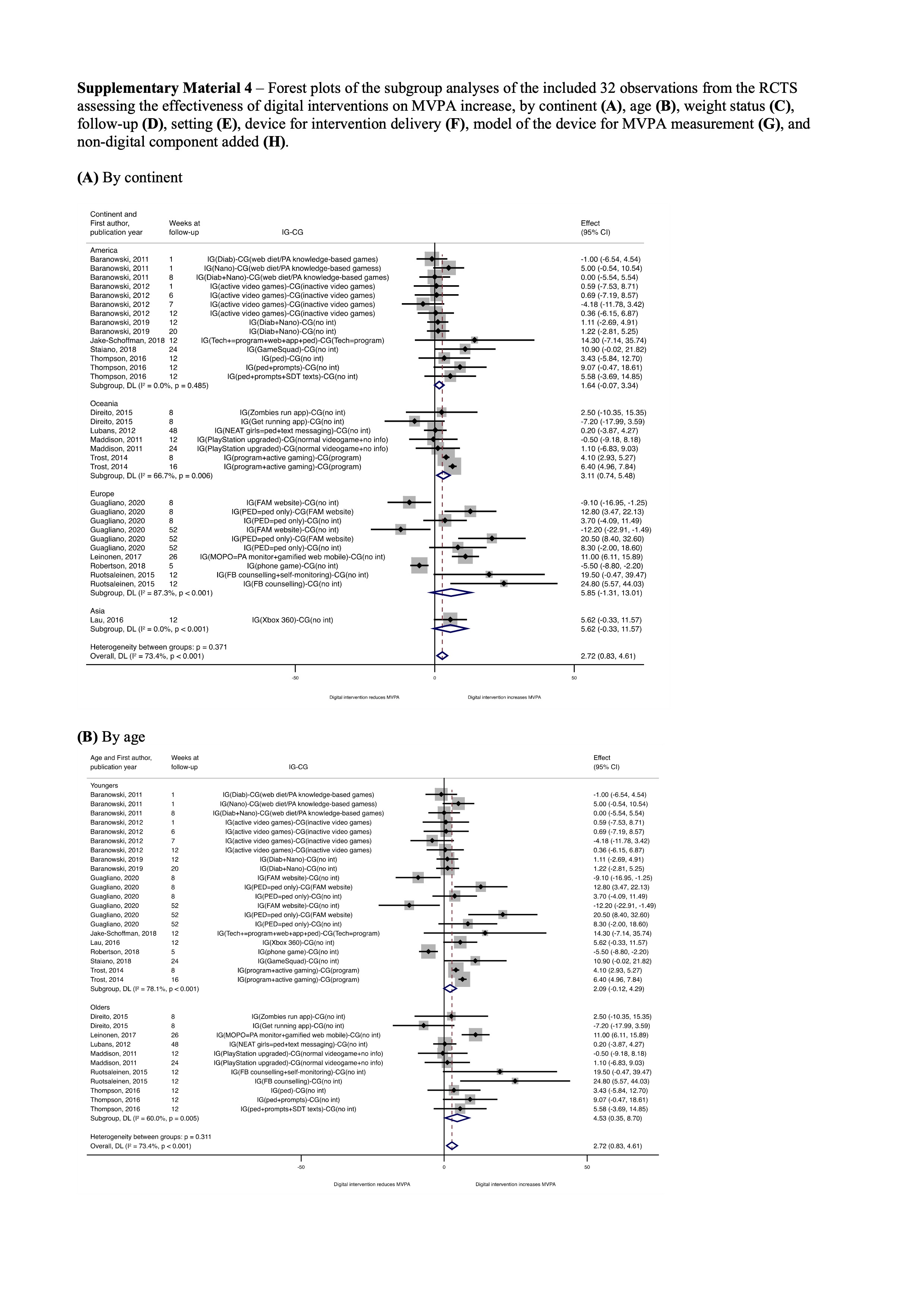

Supplement: Supplementary file 4 [file Supplementaryfile4.jpeg]

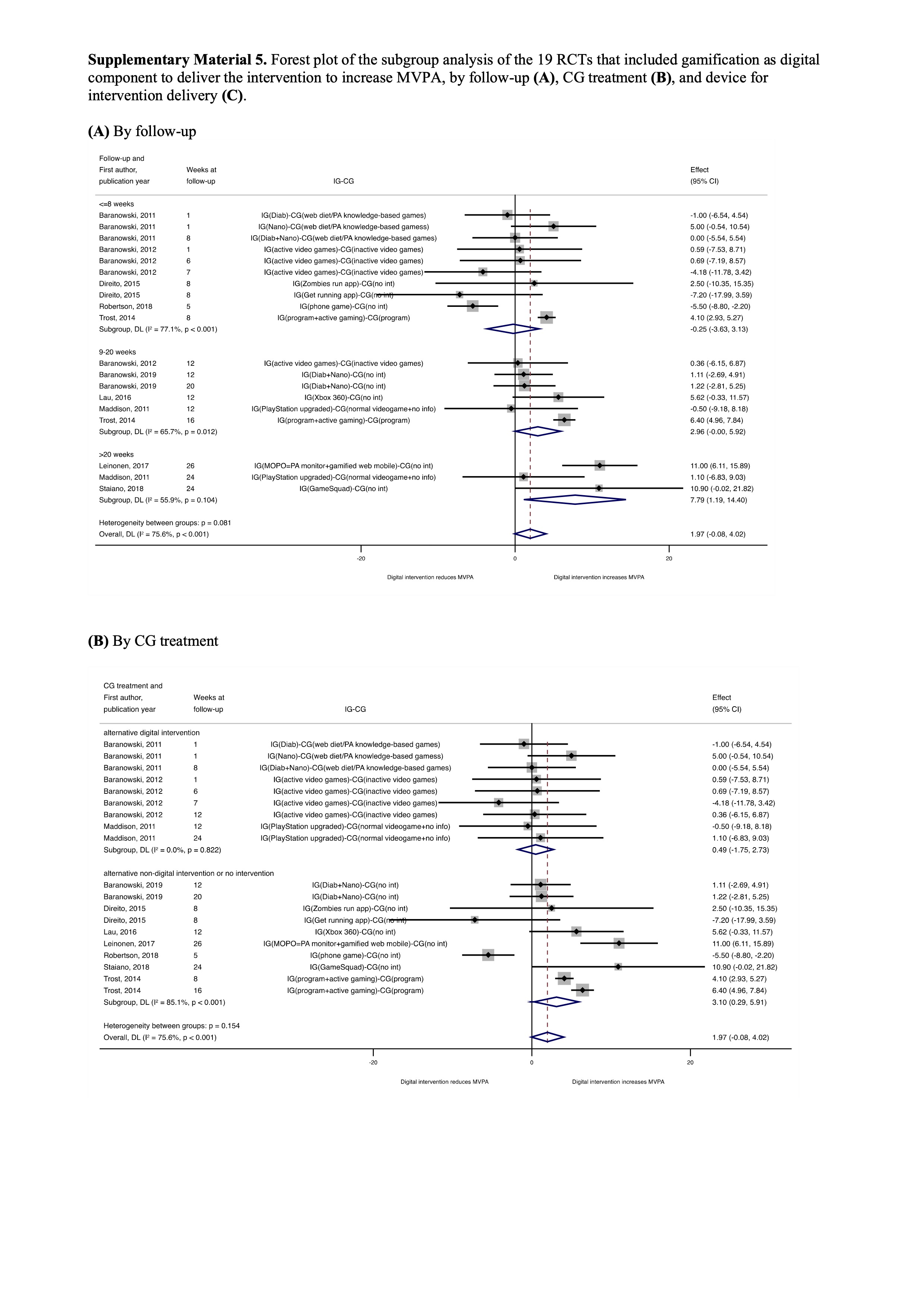

Supplement: Supplementary file 5 [file Supplementaryfile5.jpeg]
