# Supplementary material for: Meta-analyses of randomized controlled trials assessing the effect of digital tools on step count and moderate-to-vigorous physical activity in healthy children and adolescents
Source: Front Digit Health. 2026 Jun 4;8:1701301. doi: 10.3389/fdgth.2026.1701301 (PMC13275700; doi:10.3389/fdgth.2026.1701301)
Supplement: Supplementary file 3 [file Supplementaryfile3.docx]

**Supplementary Material 3**. Prediction Intervals (PI) of the effect in studies that were effective in increasing step count and MVPA.
